# Anthocyanidins and anthocyanins: colored pigments as food, pharmaceutical ingredients, and the potential health benefits

**DOI:** 10.1080/16546628.2017.1361779

**Published:** 2017-08-13

**Authors:** Hock Eng Khoo, Azrina Azlan, Sou Teng Tang, See Meng Lim

**Affiliations:** ^a^ Department of Nutrition and Dietetics, Faculty of Medicine and Health Sciences, Universiti Putra Malaysia, Selangor Darul Ehsan, Malaysia; ^b^ Research Centre of Excellence for Nutrition and Non-communicable Diseases, Faculty of Medicine and Health Sciences, Universiti Putra Malaysia, Selangor Darul Ehsan, Malaysia; ^c^ Nutritional Sciences Program, School of Healthcare Science, Faculty of Health Sciences, Universiti Kebangsaan Malaysia, Kuala Lumpur, Malaysia

**Keywords:** Anthocyanin, colorant, disease, health benefit, pigment

## Abstract

Anthocyanins are colored water-soluble pigments belonging to the phenolic group. The pigments are in glycosylated forms. Anthocyanins responsible for the colors, red, purple, and blue, are in fruits and vegetables. Berries, currants, grapes, and some tropical fruits have high anthocyanins content. Red to purplish blue-colored leafy vegetables, grains, roots, and tubers are the edible vegetables that contain a high level of anthocyanins. Among the anthocyanin pigments, cyanidin-3-glucoside is the major anthocyanin found in most of the plants. The colored anthocyanin pigments have been traditionally used as a natural food colorant. The color and stability of these pigments are influenced by pH, light, temperature, and structure. In acidic condition, anthocyanins appear as red but turn blue when the pH increases. Chromatography has been largely applied in extraction, separation, and quantification of anthocyanins. Besides the use of anthocyanidins and anthocyanins as natural dyes, these colored pigments are potential pharmaceutical ingredients that give various beneficial health effects. Scientific studies, such as cell culture studies, animal models, and human clinical trials, show that anthocyanidins and anthocyanins possess antioxidative and antimicrobial activities, improve visual and neurological health, and protect against various non-communicable diseases. These studies confer the health effects of anthocyanidins and anthocyanins, which are due to their potent antioxidant properties. Different mechanisms and pathways are involved in the protective effects, including free-radical scavenging pathway, cyclooxygenase pathway, mitogen-activated protein kinase pathway, and inflammatory cytokines signaling. Therefore, this review focuses on the role of anthocyanidins and anthocyanins as natural food colorants and their nutraceutical properties for health.

**Abbreviations**: CVD: Cardiovascular disease VEGF: Vascular endothelial growth factor

## Introduction

**Figure 1.**
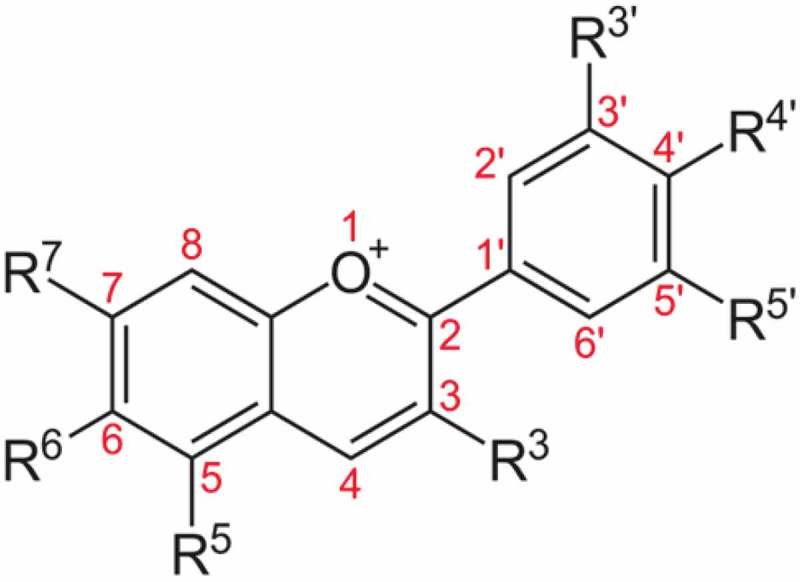
Basic anthocyanin structure.

Anthocyanins are blue, red, or purple pigments found in plants, especially flowers, fruits, and tubers. In acidic condition, anthocyanin appears as red pigment while blue pigment anthocyanin exists in alkaline conditions. Anthocyanin is considered as one of the flavonoids although it has a positive charge at the oxygen atom of the C-ring of basic flavonoid structure. It is also called the flavylium (2-phenylchromenylium) ion. The general molecular structure of anthocyanin is shown in Figure 1. The stability of anthocyanin is dependent on pH, light, temperature, and its structure [1].

Anthocyanins are commonly found in flowers and the fruits of many plants. Most of the red, purple, and blue-colored flowers contained anthocyanins. Red flowers are red hibiscus, red rose, red pineapple sage, red clover, and pink blossom. These red flowers are edible. Blue (cornflower, blue chicory, and blue rosemary) and purple (purple mint, purple passion flower, purple sage, common violet, and lavender) flowers are the common edible flowers. Some of these flowers have been traditionally used as folk medicine, as colorants, and as food. In addition to traditional usage, red, purple, and blue-colored fruits are commonly consumed for their beneficial effects. The colored pigments of anthocyanin from berries, blackcurrants, and other types of red to blue-colored fruits are strong antioxidants. Moreover, anthocyanin-rich black carrot, red cabbage, and purple potato are potential functional foods that have been consumed for prevention of diseases.

Anthocyanins found in plants have a wide range of usage. Blue, red, and purple colored pigments extracted from flowers, fruits, and vegetables are traditionally used as dye and food colorant. Besides being used as natural colorants, some of the anthocyanin-rich flowers and fruits have been traditionally used as medicine to treat various diseases. On the other hand, plant anthocyanins have been widely studied for their medicinal values. Anthocyanins possess antidiabetic, anticancer, anti-inflammatory, antimicrobial, and anti-obesity effects, as well as prevention of cardiovascular diseases (CVDs) [2]. Therefore, anthocyanins extracted from edible plants are potential pharmaceutical ingredients.

## Types of anthocyanin in plants

Anthocyanin is one of the subclasses of phenolic phytochemicals. Anthocyanin is in the form of glycoside while anthocyanidin is known as the aglycone. Anthocyanidins are grouped into 3-hydroxyanthocyanidins, 3-deoxyanthocyanidins, and O-methylated anthocyanidins, while anthocyanins are in the forms of anthocyanidin glycosides and acylated anthocyanins. The most common types of anthocyanidins are cyanidin, delphinidin, pelargonidin, peonidin, petunidin, and malvidin. Acylated anthocyanins are also detected in plants besides the typical anthocyanins. Acylated anthocyanin is further divided into acrylated anthocyanin, coumaroylated anthocyanin, caffeoylated anthocyanin, and malonylated anthocyanin.

**Figure 2.**
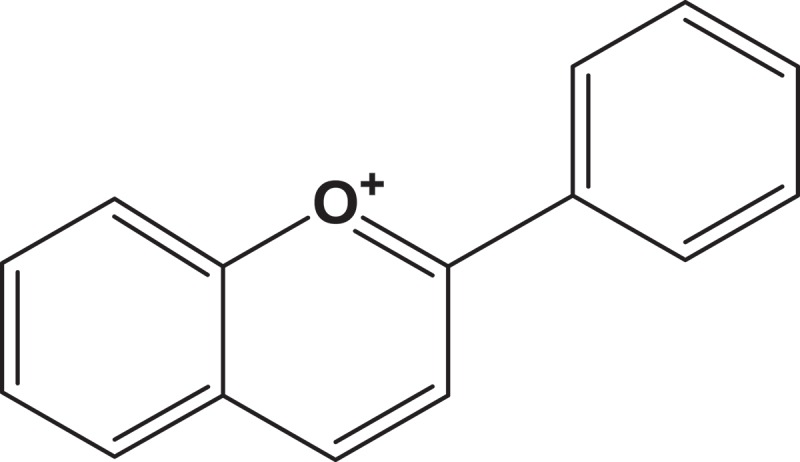
Two-dimensional structure of flavylium ion.

Anthocyanin is derived from flavonol, and it has the basic structure of flavylium ion, that is a lack of a ketone oxygen at the 4-position (Figure 2). The empirical formula for flavylium ion of anthocyanin is C_15_H_11_O^+^ with a molecular weight of 207.24724 g/mol. On the other hand, anthocyanins are the glycosylated form of anthocyanidins. The conjugated bonds of anthocyanins result in red, blue, and purple-colored plants.

**Figure 3.**
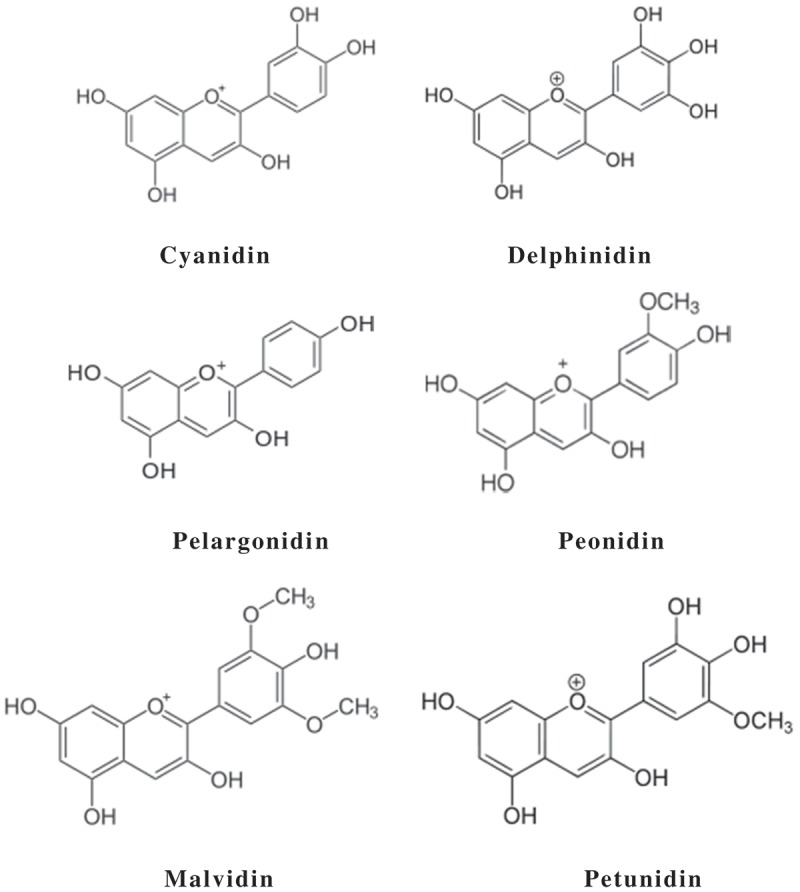
Major anthocyanidins in plants.

Cyanidin, delphinidin, pelargonidin, peonidin, malvidin, and petunidin are the most common anthocyanidins distributed in the plants. The distribution of these anthocyanidins in fruits and vegetables is 50%, 12%, 12%, 12%, 7%, and 7%, respectively [3]. Their molecular structures are shown in Figure 3. In nature, cyanidin is a reddish-purple (magenta) pigment. It is the major pigment in berries [4] and other red-colored vegetables such as red sweet potato and purple corn [5]. Delphinidin has a chemical characteristic similar to most of the anthocyanidins. It appears as a blue-reddish or purple pigment in the plant. The blue hue of flowers is due to the delphinidin pigment [6]. Pelargonidin differs from most of the anthocyanidins. In nature, it appears as red-colored pigment [7]. Pelargonidin gives an orange hue to flowers [8] and red to some of the fruits and berries [9].

Methylated anthocyanidin such as peonidin is another type of anthocyanidin abundantly found in plants. It has the visible color magenta [7]. Peonidin is abundantly found in berries, grapes, and red wines. Malvidin is another O-methylated anthocyanidin. It has a purple visible color, and is abundant in blue-colored flowers, especially Summer Wave Blue [10]. Malvidin is also the major red pigment in red wine [11]. It appears as darker dusty red in matured red wines [12]. Petunidin is a methylated anthocyanidin. It is a dark red or purple pigment that is soluble in water [7]. Petunidin has been detected in blackcurrants [13] and purple petals of flower [14].

## Color and stability of anthocyanin pigments

### Stability of anthocyanin color based on pH

Stability of anthocyanins is dependent on the type of anthocyanin pigment, copigments, light, temperature, pH, metal ions, enzymes, oxygen, and antioxidants [15]. Anthocyanidinʼs stability is also influenced by the B-ring in the anthocyanidin structure and the presence of hydroxyl or methoxyl groups [3]. These groups are known to decrease anthocyanidin stability in a solution. In this section, we discuss the stability of anthocyanin and its color changes in different pH conditions.

The color of anthocyanins is depending on the pH of the solution. This is because of the molecular structure of anthocyanins having an ionic nature [15]. In acidic condition, some of the anthocyanins appear red. Anthocyanins have a purple hue in neutral pH while the color changes to blue in an increasing pH condition. The red-colored pigments of anthocyanins are predominantly in the form of flavylium cations [7]. These anthocyanins are more stable at a lower pH solution. At lower pH, the flavylium cation formed enables the anthocyanin to be highly soluble in water. The decrease in water concentration increases the rate of deprotonation of the flavylium cation, thus reducing color stability [16]. Apart from the pH, anthocyanin-tannin polymerization could also increase the color stability at a lower pH [17].

At increasing pH conditions, colorless carbinol pseudobase and chalcone structures are formed, followed by formation of anionic quinonoidal species. This is due to the kinetic and thermodynamic competition between the hydration reaction of flavylium ion [18]. This blue quinonoidal species is unstable at lower pH. At pH 4–5, an anthocyanin solution has very little hue due to the small amount of flavylium cation and quinonoidal anion [19]. At neutral pH, resonance-stabilized quinonoid anions (color purple of anthocyanins) are formed from further deprotonation of the quinonoidal species.

The predicted pK_a_ of a flavylium cation in a buffered solution is 1–3, pK_a_ of quinonoidal base and chalcone is 4–5, and the pK_a_ of quinonoidal and chalcone monoanions is 7.5–8.0 [20]. The flavylium cation and quinonoidal base appear red, while quinonoidal monoanion and dianion can be seen as purple and blue in aqueous solution. At a lower pH solution (pH <3), cyanidin appears to be red, violet at pH 7–8; it is blue in color at a very high pH (pH >11) [21]. Peonidin (3-O-methylated anthocyanin) has cherry red hue at low pH, but its color changes to deep blue at pH 8. It is different from most of the anthocyanidins because it has higher stability at high pH than cyanidin, delphinidin, and pelargonidin.

Peonidin is also stable at high pH, therefore, the blue hue of flowers is from peonidin [22]. On the contrary, peonidin is found in red-purplish fruits at lower pH conditions [23]. Petanin (petunidin-3-[6-O-(4-O-E-p-coumaroyl-O-α-l-rhamnopyranosyl)-β-d-glucopyranoside]-5-O-β-d-glucopyranoside), is also one of the acylated anthocyanins which are stable at higher pH [18]. Acylation of anthocyanin increases the proportion of flavylium cations and helps to maintain the redness of anthocyanin pigment even at increasing pH [19].

Most of the anthocyanin pigments have a high stability in acidic conditions compared with bases, and degradation occurs at higher pH. Cyanidin and delphinidin are the examples of anthocyanidin which is stable in acidic condition. However, there has some exception. Although delphinidin is a purple-colored pigment, the blue hue of flower could be due to the delphinidin pigment in higher pH conditions [6]. At higher pH or alkaline conditions, for example, petanin is resistant to degradation even at pH 8 [18]. Due to the high stability of petanin at high pH, it is suitable for use as a colorant for herbal beverages.

### Effect of copigmentation and temperature on color change

Copigmentation and temperature are known to influence the color change of anthocyanins in a solution in addition to different pH conditions. Copigmentation of anthocyanin aglycone is referred to as a phenomenon where anthocyanidins are reinforced by metallic ions or flavonoids. Copigmentation helps to stabilize the color of the leaves, flowers, and fruits of the plant [24]. The addition of a metal ion helps stabilization of carbinol pseudo-base structures in the equilibrium mixture of delphinidin at appropriate pH values and provides a blue hue [25]. Also, color changes of flower anthocyanins are due to the copigmentation of anthocyanidins with flavonoids, which increases the color intensity of the flower [3]. In addition, glycosylation and acylation increase the color strength of anthocyanin.

Anthocyanins are less stable at higher solution temperatures. A previous study reports that heat treatment at a maximum of 35°C reduced the total anthocyanin content in the common grape to less than half the amount in control berries at 25°C [26]. At up to 40°C, the color of anthocyanin changes from red to orange although the pH of the solution was low [27]. In contrast, heat treatment of an anthocyanin-rich extract solution may not cause a degradation of anthocyanin pigments. This is because the extract commonly contains phenolic compounds that are enzymatically degraded by polyphenol oxidase. In addition, mild heat treatment of the extract to up to 50°C has been shown to inactivate the enzymatic reaction [28]. Therefore, mild heat treatment of raw materials, such as blanching, in the food processing industry can prevent oxidation of anthocyanins by polyphenol oxidase.

## Anthocyanin pigments as food colorants and additives

The use of natural colorant and additives in processed foods and beverages is important for increasing consumer acceptability of these products. Anthocyanins are some of the natural colored pigments extracted from plants, which have an attractive hue. Anthocyanins extracted from plants are red, blue, and purple pigments. These pigments are the natural colorants with low to no toxicity. Natural colorants are somehow safe to be consumed even at higher doses compared to synthetic colorants. Anthocyanins, as natural colorants, have value-added properties [29]. These properties are antioxidants, as nutraceutical and many health benefits, such as an antimicrobial effect and prevention of chronic diseases.

### Solubility of anthocyanin pigments

Anthocyanins occur as flavylium ions in grapes and wines. During digestion, the flavylium ion is converting to carbinol pseudo-base, quinoidal-base, or the chalcone at increasing pH before being absorbed into the blood system [30]. As mentioned earlier, anthocyanin pigment appears to be red in acidic conditions and blue to purple in alkaline solutions. These colored pigments are commonly extracted from flowers, berries, blackcurrant, and purple-colored fruits and vegetables. Also, water is the typical extraction medium for isolation of anthocyanin pigments. Moreover, some food processing factories use alcoholic solutions to extract anthocyanin pigments. It is because anthocyanins are soluble in both water and most of the organic solvents. On the contrary, anthocyanin is not soluble in the apolar organic solvent. It is also not stable in alkaline or neutral solutions.

At a lower pH or in an acidic condition, anthocyanidins such as cyanidin is highly soluble in water due to the formation of flavylium cation which appears as red [19]. Acidic conditions maintain the stability of flavylium ion and increase the intensity of the red hue of the anthocyanin pigment. This characteristic makes it a good candidate as a colorant, where the red anthocyanin pigment is highly stable in acidic aqueous solutions. It is best used as a colorant for red-colored beverages. Deprotonation of the flavylium ion occurs at increasing pH, where the quinoidal bases favor alcoholic solution [16]. For example, the colors of red wine can be visualized as purplish red at lower pH or bluish-violet at higher pH. The typical range of pH in red wine is 2.5–4.0 [17]. In an alcoholic solution, the red flavylium cation substantially converts to blue quinonoidal species which makes the red wine looks bluish-violet.

Anthocyanin aglycone has higher solubility in alcohol than its glucoside, whereas glycosylated anthocyanin is highly soluble in water [31]. The polyphenolic structure of anthocyanin adds a hydrophobic characteristic to it, and makes it soluble in organic solvents, such as ethanol and methanol. Besides the flavylium cation, the solubility of anthocyanidin in water could be due to the 3-hydroxyl group in the C-ring of anthocyanidin that always linked to sugar(s), which forms a stable anthocyanin in water. Among the anthocyanidins, delphinidin is the most soluble in methanol, followed by water, ethanol, and acetone [32]. As compared to delphinidin, cyanidin has lower solubility in methanol. It is because a low yield of cyanidin has been extracted from grape skins using methanol [33]. Also, delphinidin has higher solubility in water compared to malvidin because malvidin has lower polarity than delphinidin [34].

Malvidin is highly soluble in water compared with methanol and ethanol due to the static dipole moment of malvidin in water being higher than in methanol and ethanol [35]. Its solubility in water reduces with increasing degrees of acylation [12]. At a high pH condition (pH >7), syringic acid is released from the breakdown of acylated malvidin. The diffusion coefficients of both malvidin-3-glucoside and peonidin-3-glucoside in water at room temperature (25°C) are similar, even at increasing temperature [36]. Although no study compares the solubility of these six anthocyanins in water, a decreasing polarity of anthocyanidins has been reported in the order of delphinidin, cyanidin, petunidin, pelargonidin, peonidin, and malvidin [37]. In actual fact, malvidin, peonidin, and petunidin are less soluble in water compared to cyanidin, delphinidin, and pelargonidin. It could be due to these anthocyanidins have one or more hydrophobic methoxy groups at positions 3ʹ, 5ʹ, and 3ʹ and 5ʹ of the B-ring.

The solubility of anthocyanins in water increases at lower pH values where strong protonation occurs [16]. The addition of HCl to alcohol increases the solubility of anthocyanins [35]. Malonylation of anthocyanidin also enhances its solubility in water and stabilizes its structure [31]. Therefore, malonylation of anthocyanin aglycone preserves its pigment color for the use as food colorant. Nevertheless, the color of anthocyanin is greatly dependent on the number of hydroxyl groups attached to the B-ring.

### Extraction and identification of anthocyanins

The use of organic solvents such as methanol and ethanol to extract anthocyanin pigments causes a toxicity issue. Although ethanol is considered as a generally safe extraction medium, isolation of anthocyanins using water-based extraction is consider a greener way. Subcritical water-based extraction is one of the methods that have been tested for the extraction of anthocyanins from berries. This extraction technique uses acidified water (0.01% HCl, pH ~2.3) that is subjected to high temperatures between 110–160°C under a constant pressure of 40 bars [38]. It is a highly efficient technique for extraction of anthocyanins from fruit. Anthocyanin pigments also can be extracted by the addition of sulfur dioxide to water for stabilizing the anthocyanin structure with an enhanced diffusion coefficient of anthocyanin molecules through the solid [39]. This increases the solubility of anthocyanins from the plant during extraction with water.

Anthocyanins are extracted from plants as a crude mixture. For that reason, separation or isolation of specific type of anthocyanin is needed for a specific purpose. Separation and identification of anthocyanins can be done by various chromatographic methods. These include thin layer chromatography, high speed countercurrent chromatography, high-performance liquid chromatography, cellulose column chromatography, and reversed-phase ion-pair chromatography, as well as gas chromatography. In the early days, cellulose column chromatography is used for the separation of anthocyanin mixtures, but difficulties may be encountered in applying this technique in the presence of large amounts of other flavonoid materials. Purification of anthocyanin carried out by countercurrent chromatography is also too expensive to be popular [40].

Lately, macroporous adsorption resin is used in the purification of phenolic pigments because AB-8 resin is a kind of macroporous resin specially invented for purification of flavonoid [41]. In addition to separation and identification of anthocyanins, quantification of these compounds is commonly done by various chromatographic methods. High-performance liquid chromatography is the most used method in quantification of anthocyanins. However, gas chromatography has been applied in the quantification of anthocyanins [42]. Although gas chromatography is invented specifically for identification and quantification of hydrocarbon, the use of mass spectrometry enables the determination of anthocyanins using gas chromatography.

### Anthocyanins and anthocyanidin in plants

Anthocyanins are found abundant in plants, including red-purplish or red to blue-colored fruits, leaves, flowers, roots, and grains. Types of anthocyanin and anthocyanidin have been determined in fruits and vegetables. As shown in Table 1, cyanidin-3-glucoside is the most abundant anthocyanin determined in fruits and vegetables. In plants, cyanidin-3-glucoside is formed as the consequence of low pH [54]. All berries that contain glycosides of cyanidin probably do so due to the acidic nature of the berries. Malvidin, peonidin, and petunidin are not commonly found in berries. These anthocyanidins are in methylated forms, therefore, the pigments are not commonly found in red berries. A possible reason is that methylated anthocyanidin has lesser reddening effect than the non-methylated structure. Moreover, these anthocyanidins are typically detected in blue-colored fruit.

**Table 1.** Anthocyanins and anthocyanidins in fruit, vegetables, and grains.

Types of anthocyanin and anthocyanidin in fruit
Acai berry (*Euterpe oleracea* Martius) – whole fruit [43]
cya-3-glu, cyan-3-rut, del-3-gal, del-3-glu, del-3-rut, peo-3-glu
Berry (*Berberis lycium* Royle) – whole fruit [44]
cya-3,5-dihex, cya-3-gal, cya-3-glu, cya-3-lat, cya-3-rut, del-3-glu, mal-3,5-dihex, pel-3,5-diglu, pel-3-pentoxilhex, pel-3-rut, pel-hex, peo-3-rut
Bilberry (*Vaccinium myrtillus* L.) – whole fruit [45]
cy-3-ara, cya-3-gal, cya-3-glu, del-3-ara, del-3-glu, del-3-gal, mal-3-ara, mal-3-gal, mal-3-glu, peo-3-ara, peo-3-gal, peo-3-glu, pet-3-ara, pet-3-gal, pet-3-glu
Blackberry (*Rubus fruticosus* L.) – whole fruit [46,47]
cya-3-glu, cya-3-rutl del, mal, pel, pel-3-glu, peo
Blackcurrant (*Ribes nigrum* L.) – whole fruit [46]
cya-3-glu, cya-3-rut, del-3-glu, del-3-rut
Blueberry (*V. corymbosum* L.) – whole fruit [46]
cya-3-ara, cya-3-gal, cya-3-glu, del-3-ara, del-3-gal, del-3-glu, mal-3-ara, mal-3-gal, mal-3-glu, peo-3-gal, peo-3-glu, pet-3-ara, pet-3-gal, pet-3-glu
Cranberry (*V. oxycoccos* L.) – whole fruit [46]
cya-3-ara, cya-3-gal, peo-3-ara, peo-3-gal
Dabai (*Canarium odonthophyllum* Miq.) – skin [48]
cya-3-glu, cya-3-gal, cya-3-ara, cya-3-sop, cya-3-rut, del-3-glu, del-3-gal, mal-3,5-diglu
Maqui berry [*Aristotelia chilensis* (Mol.) Stuntz] – whole fruit [49]
cya-3-glu, cya-3-sam, cya-diglu, cya-sam-glu, del-3-glu, del-3,5-diglu, del-3-sam, del-3-sam-5-glu
Nitratia (*Nitraria tangutorun* Bobr.) – seed [50]
cya-3-O-(caffeoyl)-diglu, cya-3-O-(cis-p-coumaroyl)-diglu, cya-3-O-(trans-p-coumaroyl)-diglu, cya-3-diglu, del-3-O-(p-coumaroyl)-hexose, del-3-O-(caffeoyl)-diglu, pel-3-O-(*p*-coumaroyl)-diglu, pel-3-*O*-diglu
Oregon grape (*Mahonia aquifolium* (Pursh) Nutt) *–* whole fruit [51]
cya-3-glu, cya-3-rut, del-3-glu, del-3-rut, mal-3-glu, pel-3-glu, peo-3-glu
Pomegranate (*Punica granatum* cv. Mollar de Elche) *–* edible flesh [52]
cya-3,5-diglu, cya-3-glu, cya-pen, del-3,5-di-glu, del-3-glu, pel-3,5-di-glu, pel-3-glu
Raspberry (*Rubus idaeus* L.) – whole fruit [46]
cya-3-glu, cya-3-rut, cya-3-sop
Red grape (*Vitis vinifera* L.) from different cultivars – whole fruit [53]
cya-3-O-glu, del-3-O-glu, mal-3-O-acetylglu, mal-3-O-glu, mal-3-p-coumarylglu, peo-3-O-acetylglu, peo-3-O-glu, peo-3-p-coumarylglu, pet-3-O-glu

Types of anthocyanin and anthocyanidin in vegetables and grains
Black carrots (*Daucus carota* ssp. *sativus* var. *atrorubens* Alef.) [55]
cya-3-xylosyl-glucosyl-gal, cya-3-xylosyl-gal, cya-3-xylosyl-glucosyl-gal-coumaric acid, cya-3-xylosyl-glucosyl-gal-ferulic acid, cya-3-xylosyl-glucosyl-gal-sinapic acid
Black soybean [*Glycine max* (L.) Merrill] [56]
cya-3-gal, cya-3-glu, del-3-glu, peo-3-glu.
Purple corn (*Zea mays* L.) [57]
cya-3-(6″-malonylglu), cya-3,5-diglu, cya-3-dimalonylglu, cya-3-glu, cya-3-malonylglu, cya-3-malonylglu, cya-3-succinylglu, pel-3-(6″-maolonylglu), pel-3-dimalonylglu, pel-3-glu, peo-3-(6″-malonylglu), peo-3-dimalonylglu, peo-3-glu
Purple sweet potato (*Ipomoea batatas* L.) [58]
cya-3-(caffeoyl sop)-5-glu, cya-3-(caffeoyl-feruloyl sop)-5-glu, cya-3-(caffeoyl-p-hydroxybenzoylsop)-5-glu, cya-3-(dicaffeoylsop)-5-glu, cya-3-(feruloyl sop)-5-glu, cya-3-(p-hydroxybenzoylsop)-5-glu, cya-3-sop-5-glu, pel-3-(caffeoyl-feruloyl sop)-5-glu, peo-3-(caffeoyl sop)-5-glu, peo-3-(caffeoyl-feruloyl sop)-5-glu, peo-3-(caffeoyl-p-coumaroyl sop)-5-glu, peo-3-(caffeoyl-p-hydroxybenzoylsop)-5-glu, peo-3-(dicaffeoylsop)-5-glu, peo-3-(feruloyl sop)-5-glu, peo-3-(feruloyl-p-coumaroyl sop)-5-glu, peo-3-(feruloyl-p-hydroxybenzoylsop)-5-glu, peo-3-(p-hydroxybenzoylsop)-5-glu, peo-3-sop-5-glu
Red cabbage (*Brassica oleracea* L. var. *capitata* L.) [59]
cya-3-glu, cya-3-rut, del-3-glu, del-3-rut, cya-3-diglu-5-glu, cya-3-(caffeoyl)(p-coumaroyl) diglu-5-glu, cya-3-(sinapoyl) diglu-5-glu, cya-3-(caffeoyl-sinapoyl) diglu-5-glu, cya-3-(p-coumaroyl)(sinapoyl) triglu-5-glu, cya-3-(feruloyl)(sinapoyl) triglu-5-glu, cya-3-(p-coumaroyl) diglu-5-glu, cya-3-(sinapoyl) diglu-5-glu, cya-3-(p-coumaroyl)(sinapoyl) diglu-5-glu, cya-3-(feruloyl)(sinapoyl) diglu-5-glu, cya-3-(sinapoyl)(sinapoyl) diglu-5-glu, cya-3-(sinapoyl) diglu-5-(sinapoyl) glu
Rice (*Oryza sativa* L. cv. Heugjinju) [60]
cya, cya-3-glu, peo-3-glu
Transgenic purple tomato (*Solanum lycopersicum* L. cv. Del/Ros1) [61]
del-3-(caffeoyl)-rut-5-glu, del-3-(feruloyl)-rut-5-glu, del-3-(trans-coumaroyl)-rut-5-glu, mal-3-(feruloyl)-rut-5-glu, mal-3-(p-coumaroyl)-rut-5-glu, pet-3-(feruloyl)-rut-5-glu, pet-3-(trans-coumaroyl)-rut-5-glu

*ara: arabinoside, cya: cyanidin, del: delphinidin, mal: malvidin, pel: pelargonidin, pen: pentoside, peo: peonidin, pet: petunidin, gal: galatoside, glu: glucoside, hex: hexoside, lat: lathyroside, rut: rutinoside, sam: sambubioside, sop: sophoroside

Petunidin is the anthocyanidin formed in most fruits. Other than the fruits and tomato as fruit vegetable, as well as flowers, petunidin is not commonly determined in purple-colored leaves, roots, and grains. Although most of these purple-colored vegetables contain petunidin and its glycosides, these pigments are not well-known for the potential health benefits. Similar to petunidin, malvidin is also one of the less popular anthocyanidins. Nevertheless, malvidin is a potent antioxidant with high bioavailability [62].

### Potential uses of anthocyanin pigments

Anthocyanins extracted from plants have been used as food additives. Food additive, E163, is one of the commercial additives derived from fruit anthocyanin such as grape skin. It is a purple food additive for use in producing purple-colored jam, confectionaries, and beverages. Recently, synthetic food dyes attracted public concern regarding safety and the adverse effect on human health, particularly neurological functions and behavioral effects. A clinical trial involving a total of 153 three-year-old and 144 eight to nine-year-old children suggested that artificial colorants that contained a mixture of sunset yellow (E110), carmoisine (E122), tartrazine (E102), ponceau 4R (E124), quinoline yellow (E104), and Allura red AC (E129) when combined in the diet with sodium benzoate (E211), resulted in a significant increase of hyperactivity in normal children and aggravated the condition as well at least up to middle childhood [63]. This finding has drawn great interest in exploring natural food colorants such as anthocyanin as a promising alternative to synthetic food dyes.

The use of anthocyanin-based colorants in yogurt drink and some mixed fruit juice is becoming more popular. Some companies did use synthetic dyes in their products. However, these synthetic dyes may be toxic if overconsumed. Recently, acylated anthocyanins are food colorants used in the food industry due to their high stability over nonacylated anthocyanins [64]. A high level of nonacylated anthocyanins are produced from certain fruits, such as elderberry and barberry, at relatively low cost. These commodities have potential as colorants for use in the food industry.

## Nutraceutical and pharmaceutical effects of anthocyanins

Anthocyanin is one of the bioactive components as nutraceutical and traditional medicine. It has been traditionally used as a phytopharmaceutical, appetite stimulant, choleretic agent, and for treatment of many other diseases. These colored pigments are potent nutraceutical or pharmaceutical ingredients. As a nutraceutical, the bioavailability of anthocyanin is the key factor for maintaining good health and for prevention of diseases.

The low bioavailability of anthocyanins causes low absorption of these compounds into the blood circulating system and a high excretion rate of anthocyanins in urine and feces, thus reducing the efficacy of anthocyanins in scavenging free radical. Anthocyanin with a high bioavailability efficiently reduces cellular lipid peroxidation, hence reducing the risk of many diseases. Until now, limited reports on the bioavailability of major anthocyanins are available for comparison. In this review, bioavailability of selected anthocyanidin and anthocyanin will be discussed.

Among the major anthocyanins, the bioavailability of cyanidin-3-glucoside and malvidin-3-glucoside is the most reported. The relative bioavailability of red wine anthocyanins has been reported previously, where the glycosides of peonidin had the highest relative bioavailability, followed by the glycosides of cyanidin, malvidin, delphinidin, and petunidin [65]. Although most red wine anthocyanins have low bioavailability, increased consumption of these anthocyanins may help to increase the efficacy. However, the side effects of overconsumption of anthocyanin remain unknown.

A study has determined the absorption of malvidin-3-glucoside after ingestion of red wine and red grape juice [62], where malvidin-3-glucoside was found in plasma and urine within 3 and 6 h of consumption of these beverages. The result also demonstrates that the bioavailability of malvidin-3-glucoside was about two times higher after consumption of red grape juice compared to red wines. Another study also found that the urine anthocyanin level in the urine of six healthy volunteers who drank 300 ml of red wine (218 mg anthocyanins) reached a peak within 6 h of wine consumption [66]. Another study reports on drinking of 11 g of elderberry concentrate containing 17.2% w/w of anthocyanins (mainly containing cyanidin-3-glucoside and cyanidin-3-sambubioside) on an empty stomach [67]. Based on the results obtained, low bioavailability of anthocyanins was suggested by the researchers.

In fact, anthocyanins are more bioavailable than previously assumed. Literature shows that the relative bioavailability of cyanidin-3-glucoside was 12.38 ± 1.38%, where 5.37 ± 0.67% was excreted in urine and 6.91 ± 1.59% in the breath within 48 h after oral ingestion [68]. The reported metabolites of cyanidin-3-glucoside after digestion by the human body are phenolic acid, and phenolic conjugates, hippuric, phenylacetic, and phenylpropenoic acids. On the other hand, delphinidin-3-rutinoside, cyanidin-3-rutinoside, delphinidin-3-glucoside, and cyanidin-3-glucoside from blackcurrant have been found directly absorbed into the human blood circulating system and their glycosylated forms are excreted into urine [69]. This observation is also supported by a previous study that after 30 minutes of consumption of anthocyanin mixture from red fruit, the absorbed anthocyanins were not metabolized into the aglycones or any other metabolite forms in the human body [70].

The potential health benefits of anthocyanins are summarized in Tables 2 and 3. These health benefits are antioxidative effects, antiangiogenesis, prevention of CVD, anticancer, antidiabetes, improved visual health, anti-obesity, antimicrobial, and nueroprotection. The mechanisms of the action of anthocyanidin and anthocyanin in disease prevention are discussed in the next section.

**Table 2.** Prevention of chronic diseases using plant anthocyanins.

Health benefits of anthocyanins	References
***Cardiovascular disease***	
Inhibited platelet aggregation (*in vitro* antithrombotic properties)	[71]
Possessed vasorelaxation properties in isolated coronary artery rings of mature female pigs	[72]
Decreased susceptibility to ischemia-reperfusion injury and infarct size with increased myocardial antioxidant enzyme	[73]
Improved lipid profile and platelet function in healthy volunteers	[74]
***Anticancer effect***	
Suppressed cell proliferation, inflammation, and angiogenesis and induced apoptosis in esophageal tissue of rats	[75]
Demonstrated significant anti-invasive potential in breast cancer cell lines (MDA-MB-231 and MCF7)	[76]
Demonstrated anticancer effect on BALB/c nude mice bearing MDA-MB-453 cell xenografts and breast cancer cell lines (MCF-7, MDA-MB-231, and MDA-MB-453) by inducing apoptosis and suppressing angiogenesis	[77]
Inhibited cell migration and invasion, suppressed activation of rapidly accelerated fibrosarcoma (RAF), mitogen-activated protein kinase kinase (MEK) and c-Jun N-terminal kinase (JNK), and downregulated secretion of matrix metalloproteinase 2 (MMP2) and MMP9 of MDA-MB-453 cells (HER2+)	[78]
Inhibited growth of human HT-29 colon cancer cells, increased expression of tumor suppression genes (p21WAF1 and p27KIP1) and decreased cyclooxygenase-2 gene expression	[79]
Reduced colonic aberrant crypt foci, colonic cellular proliferation and COX-2 mRNA expression in rats.	[80]
Suppressed formation of aberrant crypt foci in colons of CF-1 mice	[81]
Promoted apoptosis in benign prostatic hyperplasia rats	[82]
Possessed anti-invasive effect on human hepatoma Hep3B cells and inhibited matrix metalloproteinase MMP-2 and MMP-9 gene expression	[83]
Inhibited Akt-mTOR signalling thereby inducing maturation of acute myeloid leukaemia cells, besides inducing apoptotic players such as TRAIL in cancer systems	[84]
***Diabetes***	
Ameliorated hyperglycemia and insulin sensitivity via activation of AMP-activated protein kinase in diabetic mice	[85]
Improved dyslipidemia, enhanced antioxidant capacity, and prevented insulin resistance in human subjects with type 2 diabetes	[86]
Alleviated glomerular angiogenesis of diabetic kidneys by attenuating the induction of VEGF and HIF-1α in studied mice	[87]
Ameliorated renal apoptosis in diabetic nephropathy mice via activation of AMP-activated protein kinase (AMPK) which eventually inhibit oxidative stress and lipotoxicity.	[88]
Activated adipose tissue-derived adiponectin to defend against diabetes-related endothelial dysfunction in mice	[89]

**Table 3.** Other health benefits of plant anthocyanins.

Health benefits of anthocyanins	References
***Visual health***	
Improved visual function in patients with normal tension glaucoma	[90]
Prevented impairment of photoreceptor cell function during retinal inflammation	[91]
Decreased lens opacity together with the decreased MDA level	[92]
Suppressed cell death of HLE-B3 (lens epithelial cell line) under H_2_O_2_-induced oxidative stress	[93]
Prevented retinal degeneration induced by N-methyl-N-nitrosourea	[94]
Increased ocular blood flows but no significant changes on intraocular pressure	[95]
***Anti-obesity***	
Improved weight gain and lipid profile on obese rats.	[96]
Suppressed body weight gain and improved blood lipid profile in high-fat diet induced rats	[97]
Ameliorated obesity in high-fat-fed C57BL/6 mice	[98]
Up-regulate adipocytokine secretion and gene expression in isolated rat adipocytes	[99]
Suppressed weight gain, fat tissue gain and other metabolic disorders	[100]
***Antimicrobial***	
Possessed antimicrobial activity through damaging and destroying the cell wall, membrane and intercellular matrix	[101]
Showed antibacterial activity with the highest sensitivity to *Aeromonas hydrophilia* and *Listeria innocua*	[102]
Possessed antibacterial effects towards *Enterococcus faecium* resistant to vancomycin, *Pseudomonas aeruginosa, Staphylococcus aureus* and *Escherichia coli*	[103]
Inhibited gram-negative bacteria but not on gram-positive bacteria	[104]

### Antioxidants

The health and therapeutic effects of anthocyanin are mainly contributed by its antioxidative activities. As reported in the literature [105], anthocyanin chalcones and quinoidal bases with a double bond conjugated to the keto group are efficient antioxidants in scavenging free radicals. Also, the glycosylated B-ring structure of anthocyanin contributes to the high antioxidant activity, where ortho-hydroxylation and methoxylation substantially increase the antioxidant activity [106].

In fact, anthocyanidin has a higher ORAC value than anthocyanin. One of the possible reasons is anthocyanin aglycone is very unstable and highly reactive [107]. Anthocyanin, with the addition of an extra sugar at position C-3 in the heterocyclic C-ring, has lower antioxidant activity than the anthocyanidin with a single sugar molecule [108]. Acylation of anthocyanin with phenolic acid has a significant increase in antioxidant activity [109]. Diacylation of the anthocyanin markedly increases the antioxidant activity but 5-glycosylation leads to a reduction in the activity [107].

A previous study reports the antioxidant activity of malvidin-3-glucoside that was determined by metal-catalyzed lipid peroxidation models in comparison with other antioxidants [30]. The result shows that the quinoidal-base and pseudo-base of malvidin-3-glucoside significantly inhibited peroxidation of linoleate by myoglobin compared with catechin. In the presence of hydrogen peroxide-activated myoglobin, malvidin-3-glucoside had the highest antioxidant activity, followed by catechin, malvidin, and resveratrol. In term of glycosylated anthocyanin, addition of an extra glucose to cyanidin-3-xylosyl-galactoside forms cyanidin-3-xylosyl-glucosyl-galactoside with an ORAC value lower than the anthocyanin without addition of an extra sugar [107]. Acylation of malvidin-3-glucoside with p-coumaric acid has antioxidant activity assessed by linoleic acid oxidation higher than the non-acylated counterpart [109].

Anthocyanins have many other therapeutic effects in addition to their antioxidant activities. As an active pharmaceutical ingredient, anthocyanin pigment, such as delphinidin, has been patented for several therapeutic effects. Delphinidin is well-known for combating melanoma cells [110], as well as antimicrobial effects, such as curing *Staphylococcus aureus* infection [111]. It has also been used as the source of antiphlogistic or immunosuppressive active ingredients [112].

Literature shows that anthocyanins extracted from plants have antioxidative properties. Pelargonidin-3-glucoside, cyanidin-3-glucoside, and delphinidin-3-glucoside isolated from *Phaseolus vulgaris* L. (black bean) seed coat, as well as their standard aglycones, have strong antioxidative activity in a liposomal system and reduced formation of malondialdehyde by UVB irradiation [113]. The study also indicates that delphinidin and delphinidin-3-glucoside had the highest inhibitory effect on lipid peroxidation and O2•− scavenging activity. On the contrary, pelargonidin had the highest inhibitory effect on hydroxyl radical scavenging activity.

On the other hand, a study demonstrates that cyanidin and cyanidin-3-glucoside have the highest inhibitory effect on copper (II)-induced low-density lipoprotein (LDL) oxidation compared with the other phenolic acids, anthocyanins, and anthocyanin aglycones, whereas delphinidin has intermediate efficacy [114]. Comparing the result from both studies, the second study does not determine the efficacy of pelargonidin to inhibit lipid peroxidation.

### Angiogenesis and development of diseases

Endothelial cells are the main cells involved in the angiogenesis process. Disturbances in physiologic angiogenesis can contribute to various human diseases, including CVDs, cancer, and diabetic complications such as diabetic retinopathy and nephropathy [115]. Normal angiogenesis depends on the intricate balance between angiogenic (VEGF, FGF2-fibroblast growth factor, TGF-β-transforming growth factor, and angiopoietin) and antiangiogenic (angiostatin, endostatin, and thrombospondins) factors [116].

The antiangiogenic effect of anthocyanins has been reported by several studies. Anthocyanin-rich extracts of several berries (wild blueberry, bilberry, cranberry, elderberry, and strawberry) significantly suppress hydrogen peroxide and TNF-α-induced vascular endothelial growth factor (VEGF) expression in HaCaT cells (human keratinocytes) [117]. Bilberry anthocyanidins (delphinidin, cyanidin, and malvidin) are also reported to inhibit VEGF-induced tube formation in a co-culture of human umbilical vein endothelial cells and fibroblasts [118].

Anthocyanin-rich purple corn extract attenuates endothelial expression of VEGF and hypoxia inducible factor (HIF)-1α, as well as to induce endothelial marker of platelet endothelial cell adhesion molecule-1 and integrin β3 induced by high glucose condition in human renal mesangial and endothelial cells [87]. Also, glomerular angiogenesis in the diabetic kidneys of db/db mice is disrupted by weakening the induction of VEGF and HIF-1α *in vivo*. The purple corn extract also diminishes the mesangial and endothelial induction of angiopoietin proteins under hyperglycemic conditions. These findings suggest that anthocyanin-rich purple corn extract antagonizes glomerular angiogenesis in high blood glucose condition through disturbing the Angpt-Tie-2 ligand-receptor system linked to the renal VEGF receptor-2 signaling pathway.

### Cardiovascular health

Epidemiological studies show the relationships between anthocyanin-rich foods and CVDs, as well as the relationship between total anthocyanin intake and risk of developing these cardiovascular-related diseases. Anthocyanins also demonstrate *in vitro* anti-thrombotic effect [71]. The anti-thrombotic effect is supported by another study that anthocyanin-containing maize seed (20% seed in the diet) fed rats for eight weeks are less susceptible to ischemia-reperfusion injury and reduction of infarct size with increased myocardial antioxidant enzyme [73]. Also, Bell and Gochenaur [72] reveal that anthocyanin-rich extracts of chokeberry and bilberry, but not elderberry, possess vasorelaxation properties. Moreover, there is also no alteration of coronary response to nitric oxide which is a potent vasodilator agent.

In a clinical trial, the researchers suggest that consumption of anthocyanin-rich strawberries for one month improves lipid profile and platelet function in healthy volunteers [74]. Nonetheless, the effects may be attributed to the presence of non-anthocyanin compounds in strawberries, such as vitamin C and phenolic compounds. Moreover, the study should have control groups for comparison. However, Curtis et al. [119] indicate the consumption of 500 mg/day of elderberry extract for 12 weeks is ineffective in reducing the risk of CVD in healthy postmenopausal women. There is also no change in metabolic processing following 12 weeks of elderberry intake compared with acute intake [120].

### Anticancer

Anthocyanins have been extensively studied for their anticancer properties, as well as antiangiogenesis, based on *in vitro* and cell culture studies, and animal models. Angiogenesis is the key for cancer development, where it is an important step in the transition of tumors from a benign state to a malignant one. In cancer prevention, antiangiogenesis is the process that prevents formation of new blood vessels that supply oxygen to the tumor cells. Several phytochemicals, including flavonoids and anthocyanins, are potential antiangiogenic agents.

Anthocyanins have been extracted and isolated from different plant sources for investigating their anticancer ability on esophagus, colon, breast, liver, hematological, and prostate cancers. The evidence from a previous study shows that 5% whole freeze-dried black raspberries and the anthocyanin-rich fraction supplemented to N-nitrosomethylbenzylamine-induced F344 rats have chemopreventive potential, where the treatment groups inhibit cell proliferation, inflammation, angiogenesis, and induce apoptosis in both preneoplastic and papillomatous esophageal tissues [75]. Thus anthocyanins have chemoprophylaxis potential.

Blueberry anthocyanins and anthocyanin-pyruvic acid adduct extracts (250 μg/ml) demonstrated anti-invasive potential in both breast cancer cell lines, MDA-MB-231 and MCF7 [76]. The extracts inhibited proliferation of cancer cell by acting as chemoinhibitors. The anthocyanin-pyruvic acid adduct extract has a better effect in MDA-MB-231, suggesting an effect independent of estrogen receptors. In addition to blueberry anthocyanins, anthocyanin-rich extracts (50 µg monomeric anthocyanin/ml) from chokeberry result in 60% growth inhibition of human HT-29 colon cancer cells within 24 h exposure, increase expression of tumor suppression genes (p21WAF1 and p27KIP1) and a 35% decrease in the cyclooxygenase-2 gene expression. As expected, the extracts have no obvious growth inhibition on normal colonic cell.

In another study, supplementation of anthocyanin-rich extracts of bilberry, chokeberry, and grape (containing 3.85 g anthocyanins per kg diet) for 14 weeks significantly reduced azoxymethane-induced aberrant crypt foci by 26–29% in 3–4 week-old male-specific pathogen-free F344 rats [80]. This reduction is associated with reduced cell proliferation and decreased expression of the COX-2 gene. The result also shows that the urinary 8-OHdG levels were similar among rats fed with different diets.

Dietary anthocyanin-enriched purple-fleshed sweet potato clone P40 significantly suppresses formation of aberrant crypt foci in the colons of female CF-1 mice coincided with a greater expression of apoptotic caspase-3 in the colon mucosal epithelial cells [81]. The observation suggests that anthocyanin-enriched sweet potato P40 has a protective effect against colorectal cancer by inducing cell-cycle arrest, anti-proliferative, and through apoptotic mechanisms. Another study also proves that anthocyanin extract (2 and 5 mg/ml) of purple potato induces maturation of acute myeloid leukemia cells via TNF-related apoptosis-inducing ligand [84]. Moreover, the less common anthocyanin source from vine was reported having anti-invasive property in human hepatoma Hep3B cells in a cancer study [83].

Similarly, anthocyanin-enriched black rice extract has an anticancer effect on breast cancer cells. The extract inhibited growth of breast cancer cell lines MCF-7 (ER+, HER2/neu−), MDA-MB-231 (ER−, HER2/neu−), and MDA-MB-453 (ER−, HER2/neu+) and induces apoptosis in MDA-MB-453 cells by depolarizing mitochondrial membrane potential and releasing cytochrome C into the cytosol, and thus triggered programmed cell death through apoptosis [77]. Oral administration of the same extract (100 mg/kg/day) to BALB/c nude mice bearing MDA-MB-453 cell xenografts significantly reduced tumor growth and suppresses angiogenesis by lowering the expression of angiogenesis factors matrix metallopeptidase-9, matrix metallopeptidase-2, and urokinase plasminogen activator in the tumor tissue. The results from both *in vitro* and *in vivo* studies suggest that anthocyanin-enriched black rice extract exhibits anticancer capability against human breast cancer cells by inducing cell apoptosis and suppressing angiogenesis.

In another study, black rice anthocyanins suppress metastasis in breast cancer cells by targeting the mitogen-activated protein kinase pathway [78]. The anthocyanins inhibited migration and invasion of MDA-MB-453 cells (HER2+), suppressed activation of rapidly accelerated fibrosarcoma, mitogen-activated protein kinase (MEK), and c-Jun N-terminal kinase (JNK), as well as downregulated secretion of matrix metalloproteinase 2 (MMP2) and MMP9. The study suggests that black rice anthocyanins suppress metastasis in breast cancer cells by targeting the RAS/RAF/MAPK (retrovirus-associated DNA sequences/rapidly accelerated fibrosarcoma/mitogen-activated protein kinase) pathway. Thus, it may be useful to treat patients at an advanced cancer stage.

### Antidiabetes

The antidiabetic effect of anthocyanins from plants has been widely studied. Anthocyanin-rich Cornus fruits have been used in traditional Chinese prescription medicines to treat diabetes [121]. Primary bioactive components reported in Cornus fruits are the glycosides of cyanidin, delphinidin, and pelargonidin [98]. Jayaprakasam et al. [98] report that cyanidin-3-glucoside and delphinidin-3-glucoside effectively aided insulin secretion from rodent pancreatic β-cells (INS-1 832/13) *in vitro* compared with the other anthocyanins and anthocyanidins studied.

Another study demonstrates that pelargonidin and pelargonidin-3-galactoside caused a 1.4-fold increase in insulin secretion at 4 mM glucose concentration representative of the normal glucose level in human [122]. The ability of the anthocyanins to induce insulin secretion is in the increasing order of pelargonidin-3-galactoside, cyanidin-3-glucoside, and delphinidin-3-glucoside. This finding demonstrates that the number of hydroxyl groups on the B-ring of anthocyanins plays a crucial role in their ability to secrete insulin. Nevertheless, cyanidin, delphinidin, pelargonidin, malvidin, and petunidin do not potentiate significant insulin secretion.

In a clinical trial of 24 weeks involving 58 diabetic patients, the subjects in the anthocyanin group consumed two anthocyanin capsules (160 mg anthocyanins) twice daily purified from bilberry and blackcurrant [86]. The results show that anthocyanin group had a significantly lower fasting plasma glucose and insulin resistance index, as well as significantly elevated serum adiponectin and β-hydroxybutyrate concentrations compared to the placebo supplementation. However, the authors did not elucidate the mechanism involved for the prevention of insulin resistance in the diabetic patients.

Bilberry anthocyanin has been reported to ameliorate hyperglycemia and insulin sensitivity via activation of adenosine monophosphate-activated protein kinase (AMPK) in type 2 diabetic mice at skeletal muscle, liver, and white adipose tissue [85]. The activation of AMPK causes upregulation of glucose transporter 4 in the skeletal muscle and white adipose tissue while inhibiting glucose production in the liver. AMPK activation in the liver also results in a significant reduction in liver and serum lipid content *via* the phosphorylation of acetyl-CoA carboxylase (ACC), upregulation of peroxisome proliferator-activated receptor alpha (PPARα), acyl-CoA oxidase, and carnitine palmitoyltransferase-1A gene expressions.

It has been reported that a reduction in AMPK activity leads to diabetic nephropathy, which is associated with increased oxidative stress and lipid accumulation. Supplementation of anthocyanin-rich Seoritae extract restores AMPK activity, activates target molecules such as ACC, sterol regulatory element-binding protein 1, and PPAR, and suppresses intrarenal lipid accumulation in kidney tissue [88]. However, the authors did not examine specific contributions of the bioactive compounds in the Seoritae extract to the observed effects and the amounts of these compounds incorporated into the kidney. It is unsure if the target of anthocyanins is only AMPK or adiponectin. During the onsets of diabetic microangiopathic, microvascular permeability and the number of leucocytes sticking to the venular endothelium are increased [123]. In db/db mice, cyanidin-3-glucoside (2 g/kg diet) increases adiponectin secretion from adipose tissue, thus it protects the mice against diabetes-related endothelial dysfunction [89]. The study also shows that cyanidin-3-glucoside supplementation for eight weeks resulted in a noticeable improvement in endothelium-dependent relaxation of aorta of the mice.

### Visual health

Anthocyanin pigments are important nutraceuticals in maintaining good vision. Anthocyanin-rich berries are traditionally known for the goodness to eyes and are often associated with night vision. Most of the berries have high anthocyanins content. Oral administration of bilberry extract (contained about 39% anthocyanins) to six weeks old C57BL/6 mice has been shown to prevent impairment of photoreceptor cell function during retinal inflammation [91]. In another study, 132 patients with normal tension glaucoma were supplemented with two anthocyanins capsules (60.0 mg anthocyanin in each capsule) from bilberry daily and have improved visual function, based on the Humphrey visual field test and minimal angle of resolution best-corrected visual acuity assessment [90].

Some other berries demonstrate a protective effect for eyesight. Blackcurrant anthocyanin supplementation (50 mg/day) for 24 months increased ocular blood flow in 19 patients with open-angle glaucoma, however, there were no significant changes in the intraocular pressure [95]. Supplementation of anthocyanins (50 mg/kg body weight) from the seed coat of black soybean to N-methyl-N-nitrosourea-induced retinal degenerative rats also prevents retinal degeneration [94], and also suppresses human lens epithelial cell death under hydrogen peroxide-induced oxidative stress by 50–200 μg/ml of the extract [93]. Anthocyanin also predominates around 70% in purple corn seed [124], where purple corn seed extract decreases lens opacity together with lower malonaldehyde levels [92].

### Anti-obesity effect

Anthocyanidin and anthocyanin pigments possess anti-obesity properties. Based on a previous study, obese mice fed a diet rich in cyanidin-3-glucoside from purple corn for 12 weeks have reduced body weight, as well as decreases in white and brown adipose tissue weights [100]. The study demonstrates that hyperglycemia, hyperinsulinemia, hyperleptinemia, and an increase in the tumor necrosis factor (TNF-a) mRNA level occurred in the obese rats are normalized when treated with purple corn diet. The purple corn also suppresses mRNA levels of enzymes involved in fatty acid and triacylglycerol synthesis and lowered sterol regulatory element binding protein-1 mRNA level in the white adipose tissue. These downregulations may contribute to a low triacylglycerol accumulation in white adipose tissue.

Obesity is strongly associated with adipocyte dysfunction. Therefore, regulation of protein secretion from adipocyte or the adipocyte-specific gene expression is one of the most important targets for prevention of obesity. Tsuda and his research team further investigated the potency of anthocyanins, particularly cyanidin and cyanidin-3-glucoside on isolated rat adipocytes for anti-obesity effect. He demonstrates that the adipocytes treated with anthocyanins have increased adiponectin and leptin secretions and upregulated adipocyte-specific gene expression without activation of PPARγ in the isolated rat adipocytes [99]. Gene expression of adiponectin is also upregulated in white adipose tissue of the anthocyanin-treated mice. The increased phosphorylation of AMPK may be associated with these changes, and the monophosphate/adenosine triphosphate ratio is significantly decreased by the administration of anthocyanins.

As previously reported by Tsuda et al., adipocyte gene expression is not thoroughly studied. A further examination of gene expression profile in isolated rat adipocytes treated with anthocyanins (100 nM cyanidin-3-glucoside or cyanidin) has been performed *in vitro* [125]. Within 24 h, a total of 633 genes and 427 genes were upregulated (1.5-fold) by the treatment of adipocytes with cyanidin-3-glucoside and cyanidin, respectively. The upregulated genes include lipid metabolism and signal transduction-related genes. However, the altered genes are partially different when comparing the cyanidin-3-glucoside and cyanidin treated groups. They also report that treatment of adipocytes with cyanidin-3-glucoside and cyanidin upregulated hormone sensitive lipase and enhanced lipolytic activity based on the microarray data. Even though the findings have identified new responsive genes with potentially important functions in adipocytes related to obesity, additional investigation is needed. *In vivo* adipocytes are not likely to be exposed to the anthocyanidin due to its instability in the culture.

Another study found that the ameliorated obese mice (C57BL/6) fed with Cornelian cherries (*Cornus mas*) containing anthocyanins (1 g/kg of high fat diet) for eight weeks had a 24% decrease in weight gain and decreased lipid accumulation in the liver, as well as a significant decrease in liver triacylglycerol concentration, independent of food intake [126]. The diet containing a mixture of delphinidin, cyanidin, and pelargonidin-3-O-galactosides. On the contrary, consumption of whole blueberry powder and isolated anthocyanins from blueberry and strawberry yields a mixed result. In addition, high-fat diet mice fed with whole blueberry powder have increased body weight and adiposity relative to high-fat-fed controls [127]. Inversely, the study shows that the obese mice fed with isolated anthocyanins from the fruits reduced weight gain and body fat, but the differences were not always statistically significant. The authors also tested the purified anthocyanins and blueberry juice for the ability to prevent obesity by preparing a dose of 0.2 mg/ml anthocyanin in drinking water (0.49 mg/mouse/day). The finding shows that consumption of the purified anthocyanins suppressed the rate of fat deposition. Also, consumption of blueberry juice (2.8 ml/mouse/day; 5.3 mg of anthocyanin/mouse/day) was not as effective as the purified anthocyanins in preventing deposition of fat in the body. Moreover, lower serum leptin concentrations had been consistently observed in the purified blueberry anthocyanins (1.0 mg/ml) fed to obese mice for 72 days, which reduces the development of obesity [128].

### Antimicrobial

Polyphenolic compounds including anthocyanins possess antimicrobial activity against a wide range of microorganisms, especially in inhibiting the growth of food-borne pathogens [129]. Anthocyanins exhibit antimicrobial activity through several mechanisms, such as induced cell damage by destroying the cell wall, membrane, and intercellular matrix [101].

Based on a previous study, maqui berry extracts had antibacterial activity with the highest sensitivity to *Aeromonas hydrophilia* and *Listeria innocua* [102]. These bacteria are commonly associated with refrigerated foods as indicators of pathogenic microorganisms or as spoilage microorganisms [130]. Côté et al. [103] report that cranberry extract had antibacterial activity towards *Enterococcus faecium* resistant to vancomycin, *Pseudomonas aeruginosa, Staphylococcus aureus*, and *Escherichia coli*. The antibacterial activity of cranberry extract is not based on its low pH, but it is believed due to the other specific bioactive components, such as anthocyanins and flavonols in cranberry extracts after pH adjusted to 7.

Anthocyanin-rich extracts, such as blueberry, raspberry, blackcurrant, and strawberry extracts, inhibit Gram-negative bacteria but not Gram-positive bacteria [104]. This variation may be due to the different structures of cell wall between Gram-negative and Gram-positive bacteria, in which the outer membrane of Gram-negative bacteria acts as a preventive barrier against hydrophobic compounds but not on hydrophilic compounds [131]. These antimicrobial activities of anthocyanin-containing extracts are possibly due to the multiple mechanisms and synergistic effects of various phytochemicals in the extracts, including anthocyanins, weak organic acids, phenolic acids, and their mixtures of different chemical forms [132]. Thus, the antimicrobial effect of chemically complex compounds instead of solely anthocyanins should be extensively analyzed. Also, anthocyanins in purple, red, and blue-colored fruits and vegetables are the main bioactives in preventing microbial infection by several mechanisms.

### Neuroprotective effect of anthocyanins

The term, ‘neuroprotection’, has been defined as the protection of nerve cells from oxidative injury and neurotoxicity, which interferes with the ischemic cascade. A neuroprotective agent is a drug or natural compound that prevents the nervous system from secondary injuries. The neuroprotective effects of anthocyanins have been evaluated based on the *in vitro* and *in vivo* studies. Most of the *in vitro* studies are performed by applying cell cultures, whereas *in vivo* studies are carried out based on animal models.

The neuroprotective findings from selected studies are reported in this review. An *in vitro* study [133] shows the neuroprotective effect of cyanidin-3-glucoside and its aglycone against hydrogen peroxide-induced oxidative stress in human neuronal cells (SH-SY5Y). The results demonstrate that SH-SY5Y cells pretreated with 100 µM cyanidin and cyanidin-3-glucoside significantly increased total antioxidant activity of membrane and cytosolic fraction from the cells; cyanidin also significantly increased the percentages of mitochondrial functioning and inhibited DNA fragmentation induced by hydrogen peroxide.

Based on a previous study, cyanidin-3-glucoside (2 mg/kg body weight) isolated from *Prunus cerasus* fruit inhibited apoptosis-inducing factor from mitochondria under oxidative stress but did not block the release of cytochrome c against permanent middle cerebral artery occlusion in the cortical neurons isolated from adult mice brain [134]. Besides the reported findings, the cyanidin-3-glucoside treated mice had brain superoxide level lower than the control (0.9% normal saline), as well as with better neurological test scores.

In another study, male Sprague-Dawley rats with traumatic spinal cord injury that received 400 mg/kg body weight of cyanidin-3-glucoside had a significantly improved blood-brain barrier score by 16.7%, platform hang by 40.0%, and hind foot bar grab by 30.8% compared to vehicle treated control, as well as significant reductions in superoxide level of the spinal cords and lesion volume in the lesion periphery, and a significant increased in motor neuron cell numbers of the anterior horn in lesion periphery [135]. The data from a mouse model of late pregnancy reveals that intraperitoneal injection of cyanidin-3-glucoside blocks ethanol-mediated glycogen synthase kinase-3β by inducing phosphorylation at serine 9 and reduces the phosphorylation at tyrosine 216 [136]. The compound also ameliorates ethanol-induced oxidative stress by inhibiting expression of malondialdehyde (MDA). The study concludes that cyanidin-3-glucoside prevents neurotoxicity of ethanol.

As reported in the literature, cyanidin-3-O-ß-d-glucopyranoside isolated from mulberry extract had a neuroprotective effect on PC12 pheochromocytoma cells through inhibiting cerebral ischemic damage induced by oxygen-glucose deprivation when the cells were exposed to hydrogen peroxide (150 µM) for 24 h [137]. The researchers also developed a mouse-brain-injury model of transient middle-cerebral artery occlusion, where the mice were supplemented with cyanidin-3-O-β-d-glucopyranoside and mulberry fruit extract. The result demonstrates a reduction in the infarct volume of the brain by 18% and 26%, respectively, and both supplemented groups had a lesser number of myeloperoxidase-positive cells than the ischemic control group in the striatum and cortex of the injured brain.

Based on the previous findings, most of the studies report the neuroprotective effects of cyanidin and its glycosides. Limited study has been done to determine the neuroprotective benefits of other anthocyanidins and anthocyanins. Kim et al. [138] show the neuroprotective effect of three major anthocyanins (a mixture of cyanidin-3-glucoside, delphinidin-3-glucoside, and petunidin-3-glucoside) isolated from black soybean, against hydrogen peroxide-induced cell death. They conclude that the human brain neuroblastoma SK-N-SH cells treated with the purified anthocyanin mixture (1–25 μg/ml) had a significant reduction in intracellular ROS level in a dose-dependent manner. The anthocyanins also inhibited ROS-dependent activation of apoptosis signal-regulating kinase 1 (ASK1)–JNK/p38 pathways, stimulated expression of heme oxygenase 1, and upregulated sialidase 1 (also known as Neu1) gene expression. Based on this evidence, anthocyanins obtained from plants have a neuroprotective effect.

## Mechanisms of action in disease prevention

Anthocyanins are the good antioxidants for preventing or reducing the risk of disease. Anthocyanins reduce the risk of several diseases that can be shown by direct and indirect pathways. Direct pathway is that the colored compounds directly reduce the risk of several chronic diseases through scavenging free radicals and thus reducing oxidative stress. The indirect pathways involve downregulation of cell proliferation and apoptosis through reduction of oxidative stress and lipid peroxidation. It is commonly known that anthocyanins are the strong antioxidants that effectively scavenge free radicals. Anthocyanins reduce the risk of CVD through improving blood lipid profile and biomarkers. A reduction in certain blood biomarkers is known to prevent CVDs. Similar to many other phenolic compounds, anthocyanins inhibit cancer cell proliferation via several pathways. One of the well-known mechanisms of action is the downregulation of cyclooxygenase (COX) enzyme activity. These enzymes catalyze the formation of leukotrienes, prostacyclins, prostaglandins (PGs), and thromboxanes [139]. Downregulation of COX enzymes, including COX-1 and COX-2, reverses cell proliferation and thus reduces the risk of cancer. Anthocyanins also inhibited tumor growth by blocking activation of the mitogen-activated protein kinase pathway. Moreover, the most commonly known pathways are cytokine signaling pathways. The analysis of structure-activity relationships among flavonoids suggests that 4-hydroxylations at positions 5, 7, 31, and 41, together with a bond at C2–C3, and the B-ring attaching at the C2 position, seem necessary for the highest expression of monocyte chemoattractant protein 1 (MCP-1) [140].

### Free-radical scavenging pathway

Free radicals are generated during oxidative stress in the cellular system. Reactive oxygen species (ROS) and reactive nitrogen species (RNS) are the typical free radicals that are produced during oxidative stress. ROS and RNS are generated in several cellular systems in the human body. ROS is typically produced in cytosol, mitochondrial, peroxisomes, endoplasmic reticulum, plasma membrane, and lysosomes, whereas RNS is produced from amino acid metabolism [141].

The most commonly known ROS signaling is through the respiratory chain. It involves an electron-transfer reaction pathway, where superoxide dismutase (SOD) enzyme produces hydrogen peroxide (H_2_O_2_) in mitochondrial. At the Complexes I and III in mitochondrial, hydroxyl radical (•OH) is generated via a Fenton reaction (H_2_O_2_ + Fe^2+^→ •OH + OH− + Fe^3+^). There are other pathways involved in the production of ROS (O_2_^•−^), such as through a α-ketoglutarate dehydrogenase complex and by several oxidoreductases in the mitochondrial [141]. A similar mechanism of action also occurs in peroxisomes and lysosomes, which involves metabolism of H_2_O_2_.

Another pathway for ROS generation is the xenobiotic metabolism in the endoplasmic reticulum. In the reticulum, oxygen (O_2_) initiates a reaction with lipophilic substrates (such as fatty acids, FH) in the presence of a reducing agent-RH2 (FH + O_2_ + RH2 → AOH + R + H_2_O). The reaction continues and thus produced ROS in the microsomes. In both the endoplasmic reticulum and plasma membrane, lipid peroxidation occurs via NADPH (nicotinamide adenine dinucleotide phosphate) formation pathways. When NADPH oxidase is activated, a large amount of O_2_^•−^ and H_2_O_2_ are generated. Therefore, the ROS produced is known to cause cell proliferation in a cellular system.

Anthocyanins, as the well-known antioxidants and free radical scavengers, are able to act as reducing agents in the electron-transfer reaction pathway. The antioxidative compounds are able to donate electrons to the free radicals with unpaired electrons [142]. Anthocyanins also scavenge free radicals through two pathways that have been hypothesized in the past decades. The first pathway is the attack of hydroxyl group(s) of the B-ring of theanthocyanin structure and the second is the attack of oxonium ion on the C-ring. Anthocyanins are some of the strongest antioxidants due to the free radical scavenging abilities via both pathways.

Literature supports the fact that the number of hydroxyl groups on the B-ring of the anthocyanin structure affects the scavenging activity of the anthocyanin molecules [143]. The number of hydroxyl groups is positively associated with the scavenging activity. Nonetheless, no study reports on the mechanism of the positive charge at the oxygen atom of the C-ring of the anthocyanin structure for scavenging of free radicals. It has also been hypothesized that the superoxide O_2_^o-^ radical favors the oxonium ion of anthocyanin [144].

### Biomarkers and mechanism for cardiovascular diseases

Oxidative damage to a cardiovascular system is typically caused by ROS and RNS. During the development of CVD, oxidative stress causes vascular inflammation. Vascular inflammation alters the levels of cellular total cholesterol (TC), LDL, high-density lipoprotein (HDL), and very low-density lipoprotein (VLDL), as well as plasma malonaldehyde and other plasma enzymes (SOD, catalase, and glutathione peroxidase) levels [145]. These molecules are the prognostic markers for CVD, also called the plasma lipid profile and antioxidant biomarkers. Oxidative free radicals also initiate inflammatory responses of vascular endothelial cells and upregulate cell adhesion molecules and chemokines.

The results obtained from a double-blind clinical trial of 150 hypercholesterolemic subjects aged 40–65 years old show that consumption of anthocyanins (total intake of 320 mg anthocyanins per day) for 24 weeks significantly reduced the serum high sensitivity C-reactive protein (−21.6%), soluble vascular cell adhesion molecule-1 (−12.3%), and plasma IL-1β (−12.8%) compared to the placebo (−2.5%, 0.4%, and −1.3%, respectively) [146]. Another double-blind, randomized, placebo-controlled trial also shows consumption of anthocyanins (160 mg anthocyanins twice daily) for 12 weeks significantly increased the serum HDL-cholesterol level (13.7%) and decreased the LDL-cholesterol level (13.6%) compared to the placebo group (2.8% and −0.6%, respectively) [147]. In addition, a meta-analysis concludes that anthocyanin supplementation to patients with dyslipidemia gave a significant reduction in serum TC, triglyceride, and LDL-cholesterol levels, as well as a significant increased in the HDL-cholesterol level [148].

### Cyclooxygenase (COX) pathway

COX-1 is essential for formation of thromboxane in blood platelets and maintaining integrity of gastrointestinal epithelium. It is expressed in most of the tissues. It is known for involvment in cell signaling, regulating angiogenesis in endothelial cells, and maintaining tissue homeostasis. COX-2, also known as prostaglandin-endoperoxide synthase 2, is recognized to be the essential enzyme that is involved in the conversion of arachidonic acid (ARA) to PGH2 (ARA → PGG2 → PGH2). The biosynthetic pathway of PGs has been reported by Ricciotti and FitzGerald [149] in their review article.

COX-1 and COX-2 initiate the conversion of ARA into PGH2, where it serves as a substrate for producing a series of specific isomerases. As reported in the literature, PGE2, PGI2, PGD2, and PGF2α are the four principal bioactive prostaglandins studied. PGE2 and PGI2 are typically expressed in the vascular smooth muscle cells, platelets, as well as brain and kidney cells. PGI2 is the key prostanoid that regulates cardiovascular homeostasis [149]. Oxidative stress increases expression of PGI2 in the vascular cells. PGI2, together with PGE2, mediates vasodilation of vascular system.

COX-2 is overexpressed in benign polyps and adenocarcinomas [150]. The role and mechanism of COX-2 in cell proliferation and cell death have been clearly explained in a review article [151]. The authors of this review article explain the roles of COX-2 in the prevention of cancer, which involve cell signaling and regulation of cell proliferation and apoptosis. They also discuss the role of PGE2 in inhibiting apoptosis in several *in vitro* models.

### Mitogen-activated protein kinase pathway

Mitogen-activated protein kinases (MAPKs) are the protein kinases involved in cell survival, such as cell proliferation, differentiation, migration, and apoptosis [152]. The types of known MAPKs are extracellular signal-regulated kinase 1 and 2 (ERK 1/2), ERK 5, JNK 1–3, and p38 protein isoforms (p38α, β, γ, and δ) [153]. Among the MAPKs, p38 MAPKs regulate the expression of many cytokines [154].

Among the six typical anthocyanins, peonidin-3-glucoside is reported to downregulate ERK 1/2 expression in the H1299 cell line [155]. Anthocyanin-rich pomegranate extract also had a positive effect on UV-B-mediated phosphorylation of MAPKs pathway in normal human epidermal keratinocytes. The study reveals that the pomegranate extract (20 µg/mL) inhibited UV-B–mediated phosphorylation of MAPK (ERK l/2, p38 protein, and JNK 1/2) in a time-dependent manner [156]. Anthocyanin extract obtained from *Vitis coignetiae* Pulliat has anti-tumor activity. The extract (≤60 µg/ml), which contains delphinidin-3,5-diglucoside, cyanidin-3,5-diglucoside, petunidin-3,5-diglucoside, delphinidin-3-glucoside, malvidin-3,5-diglucoside, peonidin-3,5-diglucoside, cyanidin-3-glucoside, petunidin-3-glucoside, peonidin-3-glucoside, and malvidin-3-glucoside, induces apoptosis of HCT-116 cells through increasing phosphorylation of p38-MAPK and ERK, as well as suppressing phosphorylation of Akt (protein kinase B) and JNK [157].

### Inflammatory cytokines signaling

Chronic inflammation is linked to progression of a disease that is characterized by excessive production of cytokines, changes in the pattern of cellular signaling, and infiltration of inflammatory cells. Similar to most of the flavonoids, anthocyanins reduce inflammation through several mechanisms to attenuate and to prevent inflammatory responses. The inflammatory cells produce several cytokines, including IL-1, IL-6, and TNF-α, that change the size and number of the cells. During the inflammation process, these cytokines activate hypothalamic-pituitary-adrenal that acts on the release of glucocorticoids from the adrenal cortex. The inflammatory cytokine IL-1 also activates p38 MAPKs during the inflammatory process [154].

An *in vivo* study shows that lipopolysaccharide-induced mice fed with 10% blueberries have reduced expressions of protein and mRNA of TNF-α and IL-6 in the serum compared to the control [158]. A randomized control trial also shows that hypercholesterolemic subjects who consumed a purified anthocyanin mixture (320 mg/day) for 24 weeks had a significantly lower level of plasma IL-1β than the placebo [146]. On the contrary, the control trial reveals no significant reduction in TNF-α level between the treatment and placebo at the end of the study (week-24). Another study also indicates that treatment of human monocytic THP-1 cells with 10 mg/ml anthocyanin-containing bilberry extract (25% anthocyanin content) significantly lowered IFN-γ-induced (100 ng/ml) expressions of MCP-1, IL-6, TNF-α, intercellular adhesion molecule 1 (ICAM-1), and T-cell-specific T-box transcription factor (T-bet) [159].

## Conclusions

Anthocyanins are colored pigments in plants that possess several health benefits. These colored pigments appear red in acidic condition and show a blue hue in alkaline solution. Acylated and copigmentated anthocyanidins have higher heat stability, thus maintain the structure even in different pH conditions. Anthocyanins are the value-added colorants that can be used for preventing several diseases, including CVDs, cancers, diabetes, some metabolic diseases, and microbial infection. These compounds also improve visual ability and have neuroprotective effect. Several mechanisms of action are reported for the anthocyanidins and anthocyanins in prevention of these diseases. In a nutshell, free-radical scavenging, changes in blood biomarkers, COX and MAPKs pathways, as well as inflammatory cytokines signaling are the typical mechanisms of action of these colored pigments in prevention of diseases.

## References

[1] Laleh GH, Frydoonfar H, Heidary R, et al The effect of light, temperature, pH and species on stability of anthocyanin pigments in four *Berberis* species. Pak J Nutr. 2006;5(1):90–21.

[2] He K, Li X, Chen X, et al Evaluation of antidiabetic potential of selected traditional Chinese medicines in STZ-induced diabetic mice. J Ethnopharmacol. 2011;137(3):1135–1142. 21798327 10.1016/j.jep.2011.07.033

[3] Castañeda-Ovando A, de Lourdes Pacheco-Hernández M, Páez-Hernández E, et al Chemical studies of anthocyanins: a review. Food Chem. 2009;113(4):859–871.

[4] Seeram NP, Momin RA, Nair MG, et al Cyclooxygenase inhibitory and antioxidant cyanidin glycosides in cherries and berries. Phytomedicine. 2001;8(5):362–369. 11695879 10.1078/0944-7113-00053

[5] Cevallos-Casals BA, Cisneros-Zevallos L. Stoichiometric and kinetic studies of phenolic antioxidants from Andean purple corn and red-fleshed sweetpotato. J Agric Food Chem. 2003;51(11):3313–3319. 12744660 10.1021/jf034109c

[6] Katsumoto Y, Fukuchi-Mizutani M, Fukui Y, et al Engineering of the rose flavonoid biosynthetic pathway successfully generated blue-hued flowers accumulating delphinidin. Plant Cell Physiol. 2007;48(11):1589–1600. 17925311 10.1093/pcp/pcm131

[7] Bąkowska-Barczak A Acylated anthocyanins as stable, natural food colorants – A review. Pol J Food Nutr Sci. 2005;14/55(2):107–116.

[8] Robinson GM, Robinson R A survey of anthocyanins. II. Biochemical J. 1932;6(5):1647. 10.1042/bj0261647 PMC1261082 16744989

[9] Jaakola L New insights into the regulation of anthocyanin biosynthesis in fruits. Trends Plant Sci. 2013;18(9):477–483. 23870661 10.1016/j.tplants.2013.06.003

[10] Tanaka Y, Tsuda S, Kusumi T Metabolic engineering to modify flower color. Plant Cell Physiol. 1998;39(11):1119–1126.

[11] Mazza G, Francis FJ Anthocyanins in grapes and grape products. Crit Rev Food Sci Nutr. 1995;35(4):341–371. 7576162 10.1080/10408399509527704

[12] Barnard H, Dooley AN, Areshian G, et al Chemical evidence for wine production around 4000 BCE in the Late Chalcolithic Near Eastern highlands. J Archaeol Sci. 2011;38(5):977–984.

[13] Slimestad R, Solheim H Anthocyanins from black currants (*Ribes nigrum* L.). J Agric Food Chem. 2002;50(11):3228–3231. 12009991 10.1021/jf011581u

[14] Yabuya T, Nakamura M, Iwashina T, et al Anthocyanin-flavone copigmentation in bluish purple flowers of Japanese garden iris (*Iris ensata* Thunb.). Euphytica. 1997;98(3):163–167.

[15] Turturică M, Oancea AM, Râpeanu G, et al Anthocyanins: naturally occurring fruit pigments with functional properties. Ann Univ Dunarea de Jos Galati. Fascicle VI: Food Technol. 2015;39(1):9–24.

[16] Coutinho IB, Freitas A, Maçanita AL, et al Effect of water content on the acid–base equilibrium of cyanidin-3-glucoside. Food Chem. 2015;172:476–480. 25442581 10.1016/j.foodchem.2014.09.060

[17] Sims CA, Morris JR A comparison of the color components and color stability of red wine from Noble and Cabernet Sauvignon at various pH levels. Am J Enol Viticult. 1985;36(3):181–184.

[18] Fossen T, Cabrita L, Andersen OM Colour and stability of pure anthocyanins influenced by pH including the alkaline region. Food Chem. 1998;63(4):435–440.

[19] Khoo HE, Chew LY, Ismail A, et al. Anthocyanins in purple colored fruits. In: Sun J, Prasad KN, Ismail A, et al., editors. Polyphenols: chemistry, dietary sources and health benefits. New York: Nova Science Publisher; 2012. 133–152.

[20] Asenstorfer RE, Iland PG, Tate ME, et al Charge equilibria and pK a of malvidin-3-glucoside by electrophoresis. Anal Biochem. 2003;318(2):291–299. 12814634 10.1016/s0003-2697(03)00249-5

[21] Torskangerpoll K, Andersen ØM Colour stability of anthocyanins in aqueous solutions at various pH values. Food Chem. 2005;89(3):427–440.

[22] Sen LT, Saito N, Yokoi M, et al An acylated peonidin glycoside in the violet-blue flowers of *Pharbitis nil* . Phytochem. 1991;30(7):2387–2390. 10.1016/0031-9422(93)85436-u 7763602

[23] Cho MJ, Howard LR, Prior RL, et al Flavonoid glycosides and antioxidant capacity of various blackberry, blueberry and red grape genotypes determined by high‐performance liquid chromatography/mass spectrometry. J Sci Food and Agr. 2004;84(13):1771–1782.

[24] Trouillas P, Sancho-García JC, De Freitas V, et al Stabilizing and modulating color by copigmentation: insights from theory and experiment. Chem Rev. 2016;116(9):4937–4982. 26959943 10.1021/acs.chemrev.5b00507

[25] Ahmadiani N Anthocyanin based blue colorants [dissertation]. Ohio: Ohio State University; 2012.

[26] Mori K, Goto-Yamamoto N, Kitayama M, et al Loss of anthocyanins in red-wine grape under high temperature. J Exp Bot. 2007;58(8):1935–1945. 17452755 10.1093/jxb/erm055

[27] West ME, Mauer LJ Color and chemical stability of a variety of anthocyanins and ascorbic acid in solution and powder forms. J Agric Food Chem. 2013;61(17):4169–4179. 23534933 10.1021/jf400608b

[28] Patras A, Brunton NP, O’Donnell C, et al Effect of thermal processing on anthocyanin stability in foods; mechanisms and kinetics of degradation. Trends Food Sci Technol. 2010;21(1):3–11.

[29] Bridle P, Timberlake CF Anthocyanins as natural food colours—selected aspects. Food Chem. 1997;58(1):103–109.

[30] Lapidot T, Harel S, Akiri B, et al pH-dependent forms of red wine anthocyanins as antioxidants. J Agric Food Chem. 1999;47(1):67–70. 10563851 10.1021/jf980704g

[31] Pérez-Gregorio RM, García-Falcón MS, Simal-Gándara J, et al Identification and quantification of flavonoids in traditional cultivars of red and white onions at harvest. J Food Compos Anal. 2010;23(6):592–598.

[32] Kumoro AC, Retnowati DS Budiyati CS. Solubility of delphinidin in water and various organic solvents between (298.15 and 343.15) K. J Chem Eng Data. 2010;55(7):2603–2606.

[33] Escribano-Bailón MT, Guerra MT, Rivas-Gonzalo JC, et al Proanthocyanidins in skins from different grape varieties. Eur Food Res Technol. 1995;200(3):221–224.

[34] Cheynier V, Arellano IH, Souquet JM, et al Estimation of the oxidative changes in phenolic compounds of Carignane during winemaking. Am J Enol Viticult. 1997;48(2):225–228.

[35] Iosub I, Kajzar F, Makowska-Janusik M, et al Electronic structure, optical and electrochemical properties of malvidin molecule extracted from grapes. Display Imaging. 2014;1:175–193.

[36] Srinivas K, King JW, Howard LR, et al Binary diffusion coefficients of phenolic compounds in subcritical water using a chromatographic peak broadening technique. Fluid Phase Equilib. 2011;301(2):234–243.

[37] Amić D, Davidović-Amić D, Trinajstić N Application of topological indices to chromatographic data: calculation of the retention indices of anthocyanins. J Chromatogr A. 1993;653(1):115–121.

[38] King JW, Grabiel RD, Wightman JD Subcritical water extraction of anthocyanins from fruit berry substrates. Proceedings of the 6th International Symposium on Supercritical Fluids; 2003 4 28–30; Lorraine, France: National Polytechnic Institute of Lorraine; 2003, p. 28–30.

[39] Ju ZY, Howard LR Subcritical water and sulfured water extraction of anthocyanins and other phenolics from dried red grape skin. J Food Sci. 2005;70(4):S270–S276.

[40] Schwarz M, Hillebrand S, Habben S, et al Application of high-speed countercurrent chromatography to the large-scale isolation of anthocyanins. Biochem Eng J. 2003;14(3):179–189.

[41] Liu Y, Liu J, Chen X, et al Preparative separation and purification of lycopene from tomato skins extracts by macroporous adsorption resins. Food Chem. 2010;123(4):1027–1034.

[42] Petersson EV Analysis of acrylamide and anthocyanins in foods. Extraction optimisation for challenging analytes. Acta Universitatis Upsaliensis. Digital Comprehensive Summaries of Uppsala Dissertations from the Faculty of Science and Technology 687. Sweden: Uppsala University; 2009.

[43] Fibigr J, Šatínský D, Solich P A UHPLC method for the rapid separation and quantification of anthocyanins in acai berry and dry blueberry extracts. J Pharm Biomed Anal. 2017;143:204–213. 28605682 10.1016/j.jpba.2017.05.045

[44] Pradhan PC, Saha S Anthocyanin profiling of *Berberis lycium* Royle berry and its bioactivity evaluation for its nutraceutical potential. J Food Sci Technol. 2016;53(2):1205–1213. 27162400 10.1007/s13197-015-2117-4 PMC4837717

[45] Müller D, Schantz M, Richling E High performance liquid chromatography analysis of anthocyanins in bilberries (*Vaccinium myrtillus* L.), blueberries (*Vaccinium corymbosum* L.), and corresponding juices. J Food Sci. 2012;77(4):C340–C345. 22394068 10.1111/j.1750-3841.2011.02605.x

[46] Lee SG, Vance TM, Nam TG, et al Evaluation of pH differential and HPLC methods expressed as cyanidin-3-glucoside equivalent for measuring the total anthocyanin contents of berries. J Food Meas Ch. 2016;10(3):562–568.

[47] Ivanovic J, Tadic V, Dimitrijevic S, et al Antioxidant properties of the anthocyanin-containing ultrasonic extract from blackberry cultivar “Čačanska Bestrna”. Ind Crops Prod. 2014;53:274–281.

[48] Khoo HE, Azlan A, Ismail A, et al Influence of different extraction media on phenolic contents and antioxidant capacity of defatted dabai (*Canarium odontophyllum*) fruit. Food Anal Methods. 2012;5(3):339–350.

[49] Brauch JE, Reuter L, Conrad J, et al Characterization of anthocyanins in novel Chilean maqui berry clones by HPLC–DAD–ESI/MS n and NMR-spectroscopy. J Food Compos Anal. 2017;58:16–22.

[50] Sang J, Sang J, Ma Q, et al Extraction optimization and identification of anthocyanins from *Nitraria tangutorun* Bobr. seed meal and establishment of a green analytical method of anthocyanins. Food Chem. 2017;218:386–395. 27719925 10.1016/j.foodchem.2016.09.093

[51] Coklar H, Akbulut M Anthocyanins and phenolic compounds of *Mahonia aquifolium* berries and their contributions to antioxidant activity. J Funct Foods. 2017;35:166–174.

[52] Cano-Lamadrid M, Trigueros L, Wojdyło A, et al Anthocyanins decay in pomegranate enriched fermented milks as a function of bacterial strain and processing conditions. LWT-Food Sci Technol. 2017;80:193–199.

[53] García-Beneytez E, Revilla E, Cabello F Anthocyanin pattern of several red grape cultivars and wines made from them. Eur Food Res Technol. 2002;215(1):32–37.

[54] Nakajima JI, Tanaka Y, Yamazaki M, et al Reaction mechanism from leucoanthocyanidin to anthocyanidin 3-glucoside, a key reaction for coloring in anthocyanin biosynthesis. J Biol Chem. 2001;276(28):25797–25803. 11316805 10.1074/jbc.M100744200

[55] Algarra M, Fernandes A, Mateus N, et al Anthocyanin profile and antioxidant capacity of black carrots (*Daucus carota* L. ssp. *sativus* var. *atrorubens* Alef.) from Cuevas Bajas, Spain. J Food Compos Anal. 2014;33(1):71–76.

[56] Ha TJ, Lee JH, Shin SO, et al Changes in anthocyanin and isoflavone concentrations in black seed-coated soybean at different planting locations. J Crop Sci Biotechnol. 2009;12(2):79–86.

[57] Lao F, Giusti MM Quantification of purple corn (*Zea mays* L.) anthocyanins using spectrophotometric and HPLC approaches: method comparison and correlation. Food Anal Methods. 2016;9(5):1367–1380.

[58] Gras CC, Nemetz N, Carle R, et al Anthocyanins from purple sweet potato (*Ipomoea batatas* (L.) Lam.) and their color modulation by the addition of phenolic acids and food-grade phenolic plant extracts. Food Chem. 2017;235:265–274. 28554635 10.1016/j.foodchem.2017.04.169

[59] Tong T, Niu YH, Yue Y, et al Beneficial effects of anthocyanins from red cabbage (*Brassica oleracea* L. var. *capitata* L.) administration to prevent irinotecan-induced mucositis. J Funct Foods. 2017;32:9–17.

[60] Kim BG, Kim JH, Min SY, et al Anthocyanin content in rice is related to expression levels of anthocyanin biosynthetic genes. J Plant Biol. 2007;50(2):156–160.

[61] Su X, Xu J, Rhodes D, et al Identification and quantification of anthocyanins in transgenic purple tomato. Food Chem. 2016;202:184–188. 26920283 10.1016/j.foodchem.2016.01.128

[62] Bub A, Watzl B, Heeb D, et al Malvidin-3-glucoside bioavailability in humans after ingestion of red wine, dealcoholized red wine and red grape juice. Eur J Nutr. 2001;40(3):113–120. 11697443 10.1007/s003940170011

[63] McCann D, Barrett A, Cooper A, et al Food additives and hyperactive behaviour in 3-year-old and 8/9-year-old children in the community: a randomised, double-blinded, placebo-controlled trial. Lancet. 2007;370:1560–1567. 17825405 10.1016/S0140-6736(07)61306-3

[64] Giusti M, Wrolstad RE Acylated anthocyanins from edible sources and their applications in food systems. Biochem Eng J. 2003;14(3):217–225.

[65] Frank T, Netzel M, Strass G, et al Bioavailability of anthocyanidin-3-glucosides following consumption of red wine and red grape juice. Can J Physiol Pharmacol. 2003;81(5):423–435. 12774848 10.1139/y03-038

[66] Lapidot T, Harel S, Granit R, et al Bioavailability of red wine anthocyanins as detected in human urine. J Agric Food Chem. 1998;46(10):4297–4302.

[67] Mülleder U, Murkovic M, Pfannhauser W Urinary excretion of cyanidin glycosides. J Biochem Biophys Methods. 2002;53(1):61–66. 12406587 10.1016/s0165-022x(02)00093-3

[68] Czank C, Cassidy A, Zhang Q, et al Human metabolism and elimination of the anthocyanin, cyanidin-3-glucoside: a 13C-tracer study. Am J Clin Nutr. 2013;97(5):995–1003. 23604435 10.3945/ajcn.112.049247

[69] Matsumoto H, Inaba H, Kishi M, et al Orally administered delphinidin 3-rutinoside and cyanidin 3-rutinoside are directly absorbed in rats and humans and appear in the blood as the intact forms. J Agric Food Chem. 2001;49(3):1546–1551. 11312894 10.1021/jf001246q

[70] Miyazawa T, Nakagawa K, Kudo M, et al Direct intestinal absorption of red fruit anthocyanins, cyanidin-3-glucoside and cyanidin-3, 5-diglucoside, into rats and humans. J Agric Food Chem. 1999;47(3):1083–1091. 10552420 10.1021/jf9809582

[71] Rechner AR, Kroner C Anthocyanins and colonic metabolites of dietary polyphenols inhibit platelet function. Thromb Res. 2005;116(4):327–334. 16038718 10.1016/j.thromres.2005.01.002

[72] Bell DR, Gochenaur K Direct vasoactive and vasoprotective properties of anthocyanin-rich extracts. J Appl Physiol. 2006;100(4):1164–1170. 16339348 10.1152/japplphysiol.00626.2005

[73] Toufektsian MC, De Lorgeril M, Nagy N, et al Chronic dietary intake of plant-derived anthocyanins protects the rat heart against ischemia-reperfusion injury. J Nutr. 2008;138(4):747–752. 18356330 10.1093/jn/138.4.747

[74] Alvarez-Suarez JM, Giampieri F, Tulipani S, et al One-month strawberry-rich anthocyanin supplementation ameliorates cardiovascular risk, oxidative stress markers and platelet activation in humans. J Nutr Biochem. 2014;25(3):289–294. 24406274 10.1016/j.jnutbio.2013.11.002

[75] Wang LS, Hecht SS, Carmella SG, et al Anthocyanins in black raspberries prevent esophageal tumors in rats. Cancer Prev Res. 2009;2(1):84–93. 10.1158/1940-6207.CAPR-08-0155 PMC3079338 19139022

[76] Faria A, Pestana D, Teixeira D, et al Blueberry anthocyanins and pyruvic acid adducts: anticancer properties in breast cancer cell lines. Phytother Res. 2010;24(12):1862–1869. 20564502 10.1002/ptr.3213

[77] Hui C, Bin Y, Xiaoping Y, et al Anticancer activities of an anthocyanin-rich extract from black rice against breast cancer cells in vitro and in vivo. Nutr Cancer. 2010;62(8):1128–1136. 21058201 10.1080/01635581.2010.494821

[78] Chen XY, Zhou J, Luo LP, et al Black rice anthocyanins suppress metastasis of breast cancer cells by targeting RAS/RAF/MAPK pathway. BioMed Res Int. 2015;2015:414250. 26649302 10.1155/2015/414250 PMC4663286

[79] Malik M, Zhao C, Schoene N, et al Anthocyanin-rich extract from *Aronia meloncarpa* E. induces a cell cycle block in colon cancer but not normal colonic cells. Nutr Cancer. 2003;46(2):186–196. 14690795 10.1207/S15327914NC4602_12

[80] Lala G, Malik M, Zhao C, et al Anthocyanin-rich extracts inhibit multiple biomarkers of colon cancer in rats. Nutr Cancer. 2006;54(1):84–93. 16800776 10.1207/s15327914nc5401_10

[81] Lim S, Xu J, Kim J, et al Role of anthocyanin-enriched purple-fleshed sweet potato p40 in colorectal cancer prevention. Mol Nutr Food Res. 2013;57(11):1908–1917. 23784800 10.1002/mnfr.201300040 PMC3980565

[82] Jang H, Ha US, Kim SJ, et al Anthocyanin extracted from black soybean reduces prostate weight and promotes apoptosis in the prostatic hyperplasia-induced rat model. J Agric Food Chem. 2010;58:12686–12691. 21121678 10.1021/jf102688g

[83] Shin DY, Lee WS, Kim SH, et al Anti-invasive activity of anthocyanins isolated from *Vitis coignetiae* in human hepatocarcinoma cells. J Med Food. 2009;12(5):967–972. 19857058 10.1089/jmf.2008.1338

[84] Bontempo P, de Masi L, Carafa V, et al Anticancer activities of anthocyanin extract from genotyped *Solanum tuberosum* L. “Vitelotte”. J Funct Foods. 2015;19:584–593.

[85] Takikawa M, Inoue S, Horio F, et al Dietary anthocyanin-rich bilberry extract ameliorates hyperglycemia and insulin sensitivity via activation of AMP-activated protein kinase in diabetic mice. J Nutr. 2010;140(3):527–533. 20089785 10.3945/jn.109.118216

[86] Li D, Zhang Y, Liu Y, et al Purified anthocyanin supplementation reduces dyslipidemia, enhances antioxidant capacity, and prevents insulin resistance in diabetic patients. J Nutr. 2015;145(4):742–748. 25833778 10.3945/jn.114.205674

[87] Kang MK, Lim SS, Lee JY, et al Anthocyanin-rich purple corn extract inhibit diabetes-associated glomerular angiogenesis. Plos One. 2013;8(11):e79823. 24278186 10.1371/journal.pone.0079823 PMC3835931

[88] Koh ES, Lim JH, Kim MY, et al Anthocyanin-rich Seoritae extract ameliorates renal lipotoxicity via activation of AMP-activated protein kinase in diabetic mice. J Transl Med. 2015;13:203. 26116070 10.1186/s12967-015-0563-4 PMC4482313

[89] Liu Y, Li D, Zhang Y, et al Anthocyanin increases adiponectin secretion and protects against diabetes-related endothelial dysfunction. Am J Physiol Endocrinol Metab. 2014;306(8):E975–E988. 24595303 10.1152/ajpendo.00699.2013

[90] Shim SH, Kim JM, Choi CY, et al Ginkgo biloba extract and bilberry anthocyanins improve visual function in patients with normal tension glaucoma. J Med Food. 2012;15(9):818–823. 22870951 10.1089/jmf.2012.2241 PMC3429325

[91] Miyake S, Takahashi N, Sasaki M, et al Vision preservation during retinal inflammation by anthocyanin-rich bilberry extract: cellular and molecular mechanism. Lab Invest. 2012;92(1):102–109. 21894150 10.1038/labinvest.2011.132

[92] Thiraphatthanavong P, Wattanathorn J, Muchimapura S, et al Preventive effect of *Zea mays* L. (purple waxy corn) on experimental diabetic cataract. BioMed Res Int. 2014;2014:507435. 24527449 10.1155/2014/507435 PMC3914321

[93] Mok JW, Chang DJ, Joo CK Antiapoptotic effects of anthocyanin from the seed coat of black soybean against oxidative damage of human lens epithelial cell induced by H_2_O_2_ . Curr Eye Res. 2014;39(11):1090–1098. 24749765 10.3109/02713683.2014.903497

[94] Paik SS, Jeong E, Jung SW, et al Anthocyanins from the seed coat of black soybean reduce retinal degeneration induced by N-methyl-N-nitrosourea. Exp Eye Res. 2012;97(1):55–62. 22387136 10.1016/j.exer.2012.02.010

[95] Ohguro H, Ohguro I, Katai M, et al Two-year randomized, placebo-controlled study of black currant anthocyanins on visual field in glaucoma. Ophthalmol. 2012;228(1):26–35. 10.1159/000335961 22377796

[96] Kwon SH, Ahn IS, Kim SO, et al Anti-obesity and hypolipidemic effects of black soybean anthocyanins. J Med Food. 2007;10(3):552–556. 17887951 10.1089/jmf.2006.147

[97] Wu T, Yu Z, Tang Q, et al Honeysuckle anthocyanin supplementation prevents diet-induced obesity in C57BL/6 mice. Food Funct. 2013;4:1654–1661. 24081320 10.1039/c3fo60251f

[98] Jayaprakasam B, Vareed SK, Olson LK, et al Insulin secretion by bioactive anthocyanins and anthocyanidins present in fruits. J Agric Food Chem. 2005;53(1):28–31. 15631504 10.1021/jf049018+

[99] Tsuda T, Ueno Y, Aoki H, et al Anthocyanin enhances adipocytokine secretion and adipocyte-specific gene expression in isolated rat adipocytes. Biochem Biophys Res Commun. 2004;316(1):149–157. 15003523 10.1016/j.bbrc.2004.02.031

[100] Tsuda T, Horio F, Uchida K, et al Dietary cyanidin 3-O-β-D-glucoside-rich purple corn color prevents obesity and ameliorates hyperglycemia in mice. J Nutr. 2003;133(7):2125–2130. 12840166 10.1093/jn/133.7.2125

[101] Pojer E, Mattivi F, Johnson D, et al The case for anthocyanin consumption to promote human health: a review. Compr Rev Food Sci Food Saf. 2013;12(5):483–508. 10.1111/1541-4337.12024 33412667

[102] Genskowsky E, Puente LA, Perez-Alvarez JA, et al Determination of polyphenolic profile, antioxidant activity and antibacterial properties of maqui [*Aristotelia chilensis* (Molina) Stuntz] a Chilean blackberry. J Sci Food Agr. 2016;96(12):4235–4242. 26781384 10.1002/jsfa.7628

[103] Côté J, Caillet S, Doyon G, et al Antimicrobial effect of cranberry juice and extracts. Food Cont. 2011;22(8):1413–1418.

[104] Puupponen-Pimiä R, Nohynek L, Meier C, et al Antimicrobial properties of phenolic compounds from berries. J Appl Microbiol. 2001;90(4):494–507. 11309059 10.1046/j.1365-2672.2001.01271.x

[105] Bors W, Heller W, Michel C, et al Flavonoids as antioxidants: determination of radical-scavenging efficiencies. Methods Enzymol. 1990;186:343–355. 2172711 10.1016/0076-6879(90)86128-i

[106] Wang H, Cao G, Prior RL Oxygen radical absorbing capacity of anthocyanins. J Agric Food Chem. 1997;45(2):304–309.

[107] Stintzing FC, Stintzing AS, Carle R, et al Color and antioxidant properties of cyanidin-based anthocyanin pigments. J Agric Food Chem. 2002;50(21):6172–6181. 12358498 10.1021/jf0204811

[108] Terahara N, Callebaut A, Ohba R, et al Acylated anthocyanidin 3-sophoroside-5-glucosides from *Ajuga reptans* flowers and the corresponding cell cultures. Phytochemistry. 2001;58(3):493–500. 11557083 10.1016/s0031-9422(01)00172-8

[109] Tamura H, Yamagami A Antioxidative activity of monoacylated anthocyanins isolated from Muscat Bailey A grape. J Agric Food Chem. 1994;42(8):1612–1615.

[110] Roewer N, Broscheit J Delphinidin for combating melanoma cells. U.S. Patent Application No. 14/651,262; 2013.

[111] Roewer N, Broscheit J Use of delphinidin against *Staphylococcus aureus*. U.S. Patent Application No. 14/389,492; 2013.

[112] Roewer N, Broscheit J Delphinidin complex as an antiphlogistic or immunosuppressive active ingredient. U.S. Patent Application No. 14/443,166; 2013.

[113] Tsuda T, Shiga K, Ohshima K, et al Inhibition of lipid peroxidation and the active oxygen radical scavenging effect of anthocyanin pigments isolated from *Phaseolus vulgaris* L. Biochemical Pharmacol. 1996;52(7):1033–1039. 10.1016/0006-2952(96)00421-2 8831722

[114] Brown JE, Kelly MF Inhibition of lipid peroxidation by anthocyanins, anthocyanidins and their phenolic degradation products. Eur J Lipid Sci Technol. 2007;109(1):66–71.

[115] Xue Y, Lim S, Bråkenhielm E, et al Adipose angiogenesis: quantitative methods to study microvessel growth, regression and remodeling in vivo. Nat Protoc. 2010;5(5):912–920. 20431536 10.1038/nprot.2010.46

[116] Goodwin AM In vitro assays of angiogenesis for assessment of angiogenic and anti-angiogenic agents. Microvasc Res. 2007;74(2):172–183. 17631914 10.1016/j.mvr.2007.05.006 PMC2692317

[117] Roy S, Khanna S, Alessio HM, et al Anti-angiogenic property of edible berries. Free Rad Res. 2002;36(9):1023–1032. 10.1080/1071576021000006662 12448828

[118] Matsunaga N, Tsuruma K, Shimazawa M, et al Inhibitory actions of bilberry anthocyanidins on angiogenesis. Phytother Res. 2010;24(S1):S42–S47. 19496063 10.1002/ptr.2895

[119] Curtis PJ, Kroon PA, Hollands WJ, et al Cardiovascular disease risk biomarkers and liver and kidney function are not altered in postmenopausal women after ingesting an elderberry extract rich in anthocyanins for 12 weeks. J Nutr. 2009;139(12):2266–2271. 19793846 10.3945/jn.109.113126

[120] De Ferrars RM, Cassidy A, Curtis P, et al Phenolic metabolites of anthocyanins following a dietary intervention study in post-menopausal women. Mol Nutr Food Res. 2014;58(3):490–502. 24170677 10.1002/mnfr.201300322

[121] He J, Giusti MM Anthocyanins: natural colorants with health-promoting properties. Annu Rev Food Sci Technol. 2010;1:163–187. 22129334 10.1146/annurev.food.080708.100754

[122] Christison GB, MacKenzie HA Laser photoacoustic determination of physiological glucose concentrations in human whole blood. Med Biol Eng Comput. 1993;31(3):284–290. 8412382 10.1007/BF02458048

[123] Valensi P, Behar A, Attalah M, et al Increased capillary filtration of albumin in diabetic patients—relation with gender, hypertension, microangiopathy, and neuropathy. Metabolism. 1998;47(5):503–507. 9591738 10.1016/s0026-0495(98)90231-1

[124] Aoki H, Kuze N, Kato Y, et al Anthocyanins isolated from purple corn (*Zea mays* L.). Foods Food Ingredients J Japan. 2002;199:41–45.

[125] Tsuda T, Ueno Y, Kojo H, et al Gene expression profile of isolated rat adipocytes treated with anthocyanins. Biochim Biophys Acta. 2005;1733(2):137–147. 15863361 10.1016/j.bbalip.2004.12.014

[126] Jayaprakasam B, Olson LK, Schutzki RE, et al Amelioration of obesity and glucose intolerance in high-fat-fed C57BL/6 mice by anthocyanins and ursolic acid in Cornelian cherry (*Cornus mas*). J Agric Food Chem. 2006;54(1):243–248. 16390206 10.1021/jf0520342

[127] Prior RL, Wu X, Gu L, et al Whole berries versus berry anthocyanins: interactions with dietary fat levels in the C57BL/6J mouse model of obesity. J Agric Food Chem. 2008;56(3):647–653. 18211017 10.1021/jf071993o

[128] Prior RL, Wilkes SE, Rogers TR, et al Purified blueberry anthocyanins and blueberry juice alter development of obesity in mice fed an obesogenic high-fat diet. J Agric Food Chem. 2010;58(7):3970–3976. 20148514 10.1021/jf902852d

[129] Cushnie TPT, Lamb AJ Antimicrobial activity of flavonoids. Int J Antimicrob Agents. 2005;26(5):343–356. 16323269 10.1016/j.ijantimicag.2005.09.002 PMC7127073

[130] Iturriaga L, Olabarrieta I, De Marañón IM Antimicrobial assays of natural extracts and their inhibitory effect against *Listeria innocua* and fish spoilage bacteria, after incorporation into biopolymer edible films. Int J Food Microbiol. 2012;158(1):58–64. 22824340 10.1016/j.ijfoodmicro.2012.07.001

[131] Helander IM, Alakomi HL, Latva-Kala K, et al Characterization of the action of selected essential oil components on Gram-negative bacteria. J Agric Food Chem. 1998;46(9):3590–3595.

[132] Cisowska A, Wojnicz D, Hendrich AB Anthocyanins as antimicrobial agents of natural plant origin. Nat Prod Commun. 2011;6(1):149–156. 21366068

[133] Tarozzi A, Morroni F, Hrelia S, et al Neuroprotective effects of anthocyanins and their in vivo metabolites in SH-SY5Y cells. Neurosci Lett. 2007;424(1):36–40. 17709193 10.1016/j.neulet.2007.07.017

[134] Min J, Yu SW, Baek SH, et al Neuroprotective effect of cyanidin-3-O-glucoside anthocyanin in mice with focal cerebral ischemia. Neurosci Lett. 2011;500(3):157–161. 21651957 10.1016/j.neulet.2011.05.048

[135] Kim KT, Nam TK, Park YS, et al Neuroprotective effect of anthocyanin on experimental traumatic spinal cord injury. J Korean Neurosurg Soc. 2011;49(4):205–211. 21607177 10.3340/jkns.2011.49.4.205 PMC3098422

[136] Ke Z, Liu Y, Wang X, et al Cyanidin-3-glucoside ameliorates ethanol neurotoxicity in the developing brain. J Neurosci Res. 2011;89(10):1676–1684. 21671257 10.1002/jnr.22689 PMC3154369

[137] Kang TH, Hur JY, Kim HB, et al Neuroprotective effects of the cyanidin-3-O-β-d-glucopyranoside isolated from mulberry fruit against cerebral ischemia. Neurosci Lett. 2006;391(3):122–126. 16181734 10.1016/j.neulet.2005.08.053

[138] Kim SM, Chung MJ, Ha TJ, et al Neuroprotective effects of black soybean anthocyanins via inactivation of ASK1–JNK/p38 pathways and mobilization of cellular sialic acids. Life Sci. 2012;90(21):874–882. 22575822 10.1016/j.lfs.2012.04.025

[139] Fitzpatrick FA Cyclooxygenase enzymes: regulation and function. Curr Pharm Des. 2004;10(6):577–588. 14965321 10.2174/1381612043453144

[140] Garcia-Alonso M, Minihane AM, Rimbach G, et al Red wine anthocyanins are rapidly absorbed in humans and affect monocyte chemoattractant protein 1 levels and antioxidant capacity of plasma. J Nutr Biochem. 2009;20(7):521–529. 18789665 10.1016/j.jnutbio.2008.05.011

[141] di Meo S, Reed TT, Venditti P, et al Role of ROS and RNS sources in physiological and pathological conditions. Oxid Med Cell Longev. 2016;2016:1–44. 10.1155/2016/1245049 PMC4960346 27478531

[142] Huang D, Ou B, Prior RL The chemistry behind antioxidant capacity assays. J Agric Food Chem. 2005;53(6):1841–1856. 15769103 10.1021/jf030723c

[143] Kongpichitchoke T, Hsu JL, Huang TC Number of hydroxyl groups on the B-ring of flavonoids affects their antioxidant activity and interaction with phorbol ester binding site of PKCδ C1B domain: in vitro and in silico studies. J Agric Food Chem. 2015;63(18):4580–4586. 25907027 10.1021/acs.jafc.5b00312

[144] De Gaulejac NSC, Glories Y, Vivas N Free radical scavenging effect of anthocyanins in red wines. Food Res Int. 1999;32(5):327–333.

[145] Reis JF, Monteiro VVS, Gomes RS, et al Action mechanism and cardiovascular effect of anthocyanins: a systematic review of animal and human studies. J Transl Med. 2016;14(1):315. 27846846 10.1186/s12967-016-1076-5 PMC5111351

[146] Zhu Y, Ling W, Guo H, et al Anti-inflammatory effect of purified dietary anthocyanin in adults with hypercholesterolemia: a randomized controlled trial. Nutr Metab Cardiovasc Dis. 2013;23(9):843–849. 22906565 10.1016/j.numecd.2012.06.005

[147] Qin Y, Xia M, Ma J, et al Anthocyanin supplementation improves serum LDL-and HDL-cholesterol concentrations associated with the inhibition of cholesteryl ester transfer protein in dyslipidemic subjects. Am J Clin Nutr. 2009;90(3):485–492. 19640950 10.3945/ajcn.2009.27814

[148] Liu C, Sun J, Lu Y, et al Effects of anthocyanin on serum lipids in dyslipidemia patients: a systematic review and meta-analysis. PloS One. 2016;11(9):e0162089. 27589062 10.1371/journal.pone.0162089 PMC5010219

[149] Ricciotti E, FitzGerald GA Prostaglandins and inflammation. Arterioscler Thromb Vasc Biol. 2011;31(5):986–1000. 21508345 10.1161/ATVBAHA.110.207449 PMC3081099

[150] Williams CS, Mann M, DuBois RN The role of cyclooxygenases in inflammation, cancer, and development. Oncogene. 1999;18(55):7908–7916. 10630643 10.1038/sj.onc.1203286

[151] Sobolewski C, Cerella C, Dicato M, et al The role of cyclooxygenase-2 in cell proliferation and cell death in human malignancies. Int J Cell Biol. 2010;2010:1–21. 10.1155/2010/215158 PMC2841246 20339581

[152] Munshi A, Ramesh R Mitogen-activated protein kinases and their role in radiation response. Genes Cancer. 2013;4(9–10):401–408. 24349638 10.1177/1947601913485414 PMC3863336

[153] Cargnello M, Roux PP Activation and function of the MAPKs and their substrates, the MAPK-activated protein kinases. Microbiol Mol Biol Rev. 2011;75(1):50–83. 21372320 10.1128/MMBR.00031-10 PMC3063353

[154] Johnson GL, Lapadat R Mitogen-activated protein kinase pathways mediated by ERK, JNK, and p38 protein kinases. Science. 2002;298(5600):1911–1912. 12471242 10.1126/science.1072682

[155] Ho ML, Chen PN, Chu SC, et al Peonidin 3-glucoside inhibits lung cancer metastasis by downregulation of proteinases activities and MAPK pathway. Nutr Cancer. 2010;62(4):505–516. 20432172 10.1080/01635580903441261

[156] Afaq F, Malik A, Syed D, et al Pomegranate fruit extract modulates UV-B–mediated phosphorylation of mitogen-activated protein kinases and activation of nuclear factor kappa B in normal human epidermal keratinocytes. Photochem Photobiol. 2005;81(1):38–45. 15493960 10.1562/2004-08-06-RA-264

[157] Shin DY, Lee WS, Lu JN, et al Induction of apoptosis in human colon cancer HCT-116 cells by anthocyanins through suppression of Akt and activation of p38-MAPK. Int J Oncol. 2009;35(6):1499. 19885574 10.3892/ijo_00000469

[158] Xie C, Kang J, Ferguson ME, et al Blueberries reduce pro-inflammatory cytokine TNF-α and IL-6 production in mouse macrophages by inhibiting NF-κB activation and the MAPK pathway. Mol Nutr Food Res. 2011;55(10):1587–1591. 21887820 10.1002/mnfr.201100344

[159] Roth S, Spalinger MR, Müller I, et al Bilberry-derived anthocyanins prevent IFN-γ-induced pro-inflammatory signalling and cytokine secretion in human THP-1 monocytic cells. Digestion. 2014;90(3):179–189. 25401758 10.1159/000366055

